# Supplementary material for: Genomic landscape and chronological reconstruction of driver events in multiple myeloma
Source: Nat Commun. 2019 Aug 23;10:3835. doi: 10.1038/s41467-019-11680-1 (PMC6707220; doi:10.1038/s41467-019-11680-1)
Supplement: Supplementary file 1 — Supplementary Information [file 41467_2019_11680_MOESM1_ESM.pdf]

Supplementary Information

Supplementary Figure

Supplementary Figure 1. Potential additional novel MM driver mutations. a) The prevalence and distribution of potential driver mutations recently reported by the Myeloma Genome Project and not extracted in our data set. Each bar represents a distinct driver gene and each bar's colour indicate its prevalence across the main MM cytogenetic sub-groups. The red labels indicate mutations that acquired a significant q value running the dNdS with restricted multiple hypothesis testing correction. Conversely the blue labels indicate the genes that did not show any significant positive selection in our analysis. b) Cancer cell fraction of the driver mutations reported by the Myeloma Genome Project and not extracted in our cohort. Note how some of these mutations are associated to myeloid malignancies or clonal hemopoiesis (i.e *IDH1*, *IDH2*, *TET2* and *DNMT3A*). Nevertheless, the high cancer cell fraction of most of these rare mutations suggest that these belong to the plasma cell clone.

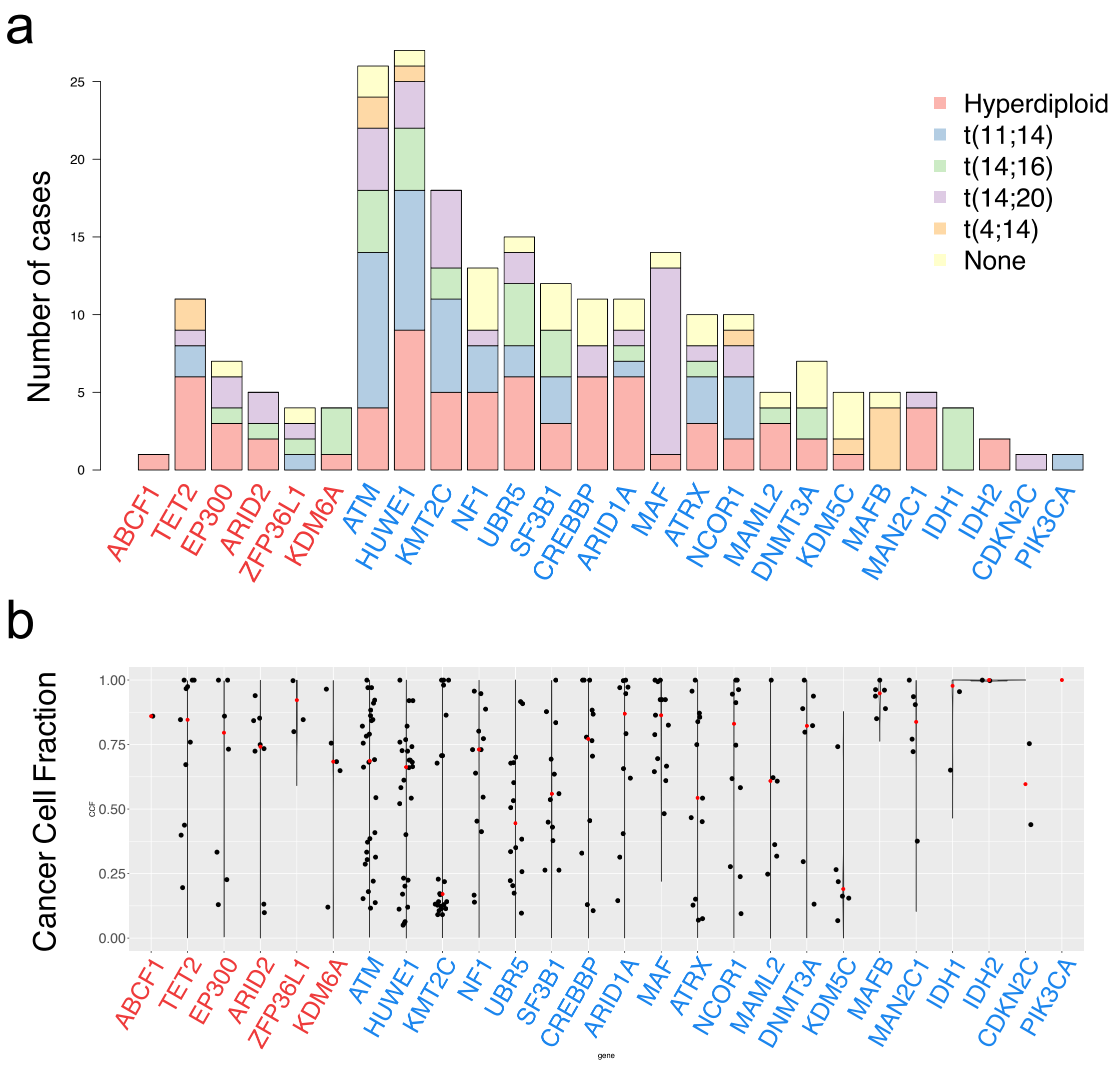
a
Number of cases
Hyperdiploid
t(11;14)
t(14;16)
t(14;20)
t(4;14)
None
ABCF1
TET2
EP300
ARID2
ZFP36L1
KDM6A
ATM
HUWE1
KMT2C
NF1
UBR5
SF3B1
CREBBP
ARID1A
MAF
ATRX
NCOR1
MAML2
DNMT3A
KDM5C
MAFB
MAN2C1
IDH1
IDH2
CDKN2C
PIK3CA
b
Cancer Cell Fraction
gene

15

Supplementary Figure 2. Driver mutations in MM. a) Cancer Cell Fraction (CCF) of each driver mutation across 834 MM patients. Each violin plot reflects the distribution of CCF across different mutated samples (dots). Driver genes were sorted according to their median CCF, which is highlighted with a red dot for each gene. b-i) Lolliplots showing the distribution of all coding mutations on *HIST1H1B* (b), *HIST1H1D* (c), *HIST1H2BK* (d), *HIST1H1E* (e), *FUBP1* (f), *MAX* (g), *ZNF292* (h) and *TBC1D29* (i). j) Pairwise genetic interactions between 69 drivers in our dataset of 724 MM patients where SVs, CNAs and driver mutations were investigated. The color of each tile reflects the odds ratio for each pair whereby pink indicates mutual exclusivity (observed, relative to the expected co-occurrence rate based on each alterations frequency) and green indicates pairs that are co-mutated (found together more frequently than would be expected by chance). White dot annotation indicates false discovery rate < 0.1; white asterisk (*) indicates a Bonferroni corrected p value <0.05.

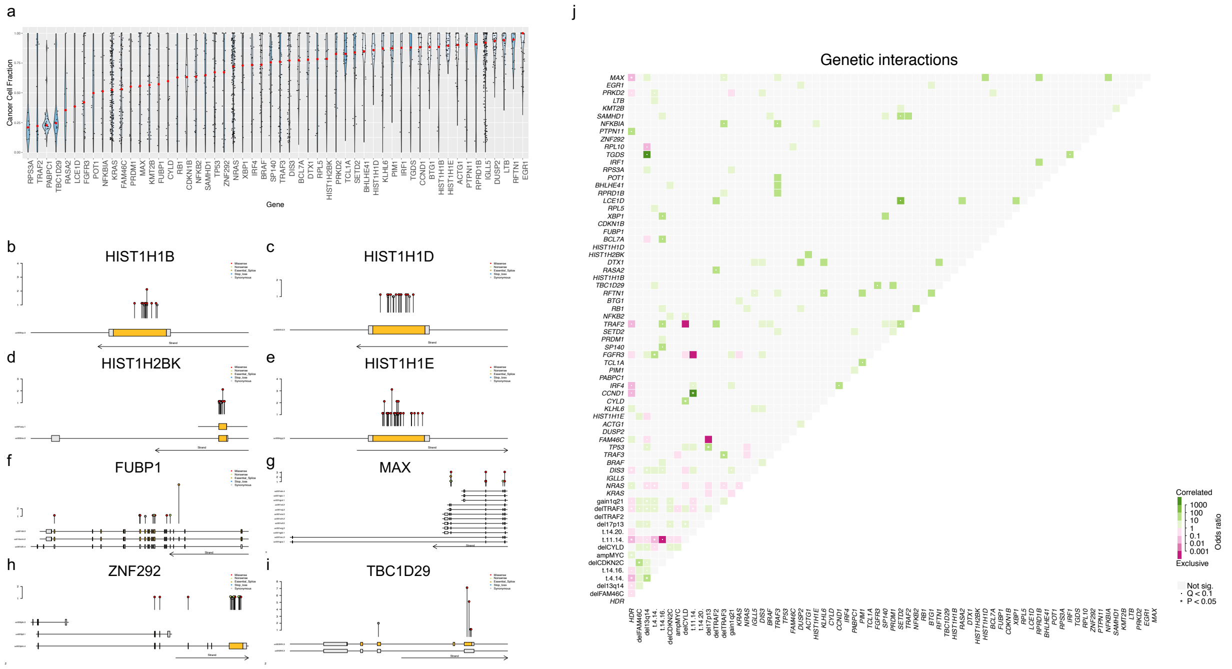

Supplementary Figure 3. Bayesian network families. a-x) Heat maps of all families observed in our Bayesian network generated from 724 MM patients.

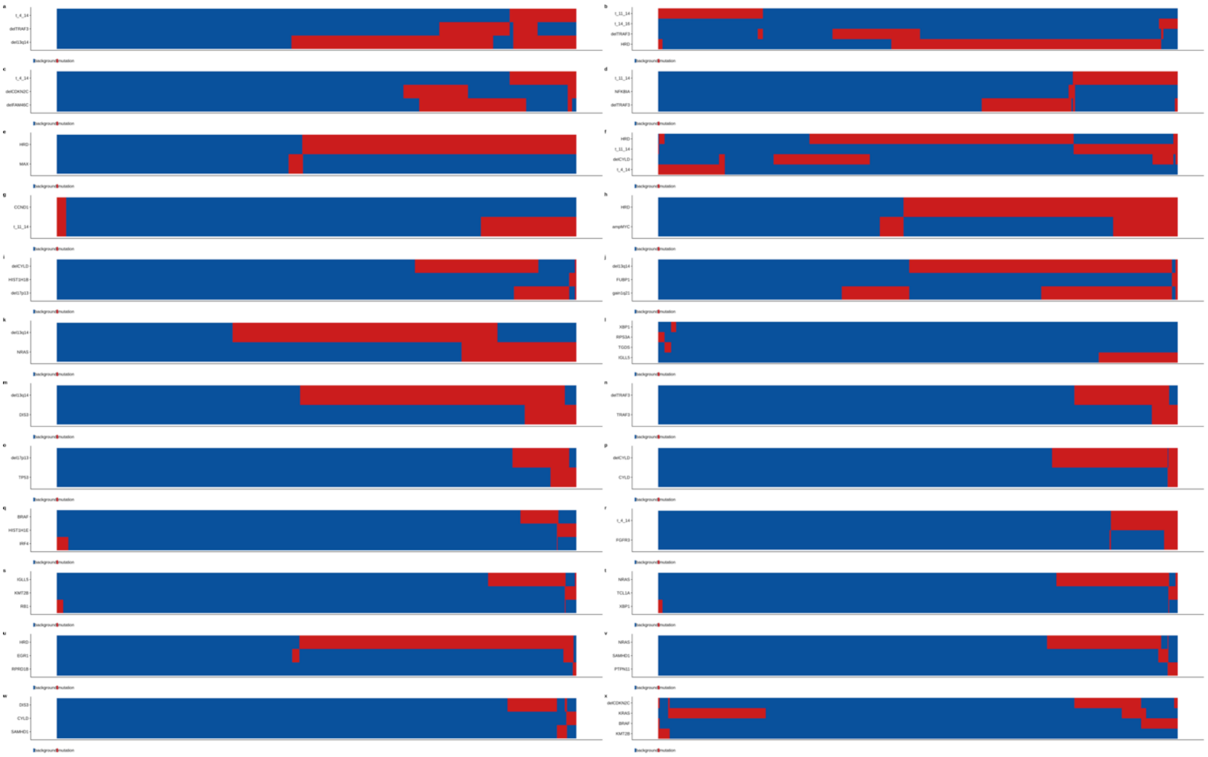

Supplementary Figure 4. SV complex event landscape in MM. a) Pie chart representing the proportion between recurrent and non-recurrent SV breakpoints across the entire series. b) Heatmap showing a mutually exclusive pattern between *IGH*, *IGK* and *IGL* translocations. Light chain translocations are always associated with the HRD status and involve the *MYC* oncogene. In turn, *MYC* can also be translocated to the IGH locus, but still correlates with an HRD karyotype. c) Prevalence of complex events across the entire series. d-f) Examples of recurrent CNAs caused by distinct unbalanced SVs. d) In PD26400a, the 1p deletion is caused by multiple inversions and two likely simultaneous translocations: one on chromosome 16 and the other on 13q. The translocation breakpoint on 13q is responsible of the centromeric deletion, suggesting that both deletions (del1p and del13q) were generated by the same event. The translocations and inversions on 1q gain are located on the non-duplicated allele (Methods), thus hampering timing correlation between this event and 1p deletion. e-f) In these two cases the 1p deletion is caused by an unbalanced inversion (e) or multiple complex inversions (f).

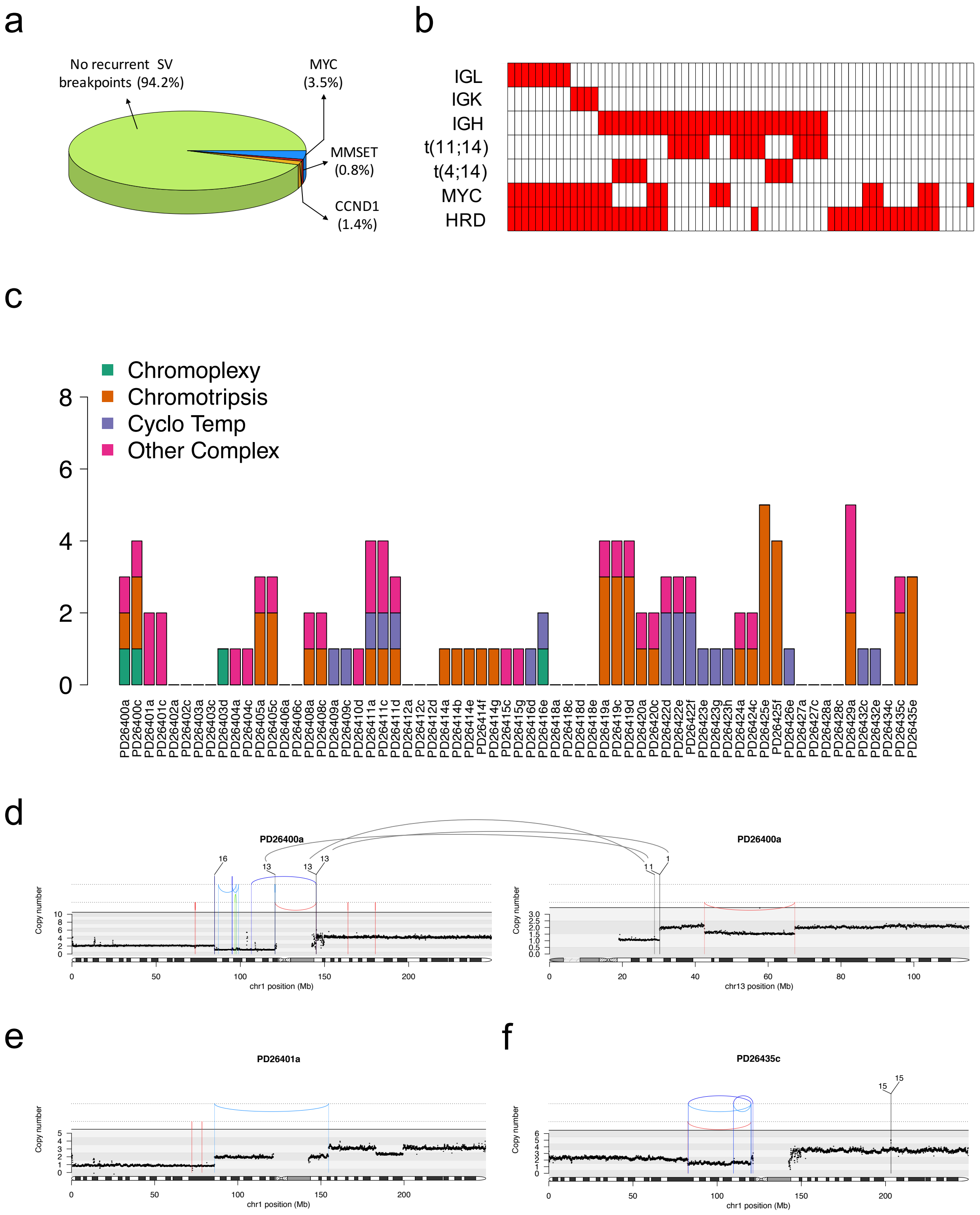

a
No recurrent SV breakpoints (94.2%)
MYC (3.5%)
MMSET (0.8%)
CCND1 (1.4%)
b
IGL
IGK
IGH
t(11;14)
t(4;14)
MYC
HRD
c
Chromoplexy
Chromotripsis
Cyclo Temp
Other Complex
0
2
4
6
8
PD26400a PD26400c PD26401a PD26401c PD26402a PD26402c PD26403a PD26403c PD26403d PD26404a PD26404c PD26405a PD26405c PD26406a PD26406c PD26408a PD26408c PD26409a PD26409c PD26410d PD26411a PD26411c PD26411d PD26412a PD26412c PD26412d PD26414a PD26414b PD26414e PD26414f PD26414g PD26415c PD26415g PD26416d PD26416e PD26418a PD26418c PD26418d PD26418e PD26419a PD26419c PD26419d PD26420a PD26420c PD26422d PD26422e PD26422f PD26423e PD26423g PD26423h PD26424a PD26424c PD26425e PD26425f PD26426e PD26427a PD26427c PD26428a PD26428c PD26429a PD26432c PD26432e PD26434c PD26435c PD26435e
d
PD26400a
Copy number
chr1 position (Mb)
PD26400a
chr13 position (Mb)
e
PD26401a
Copy number
chr1 position (Mb)
f
PD26435c
Copy number
chr1 position (Mb)

52

Supplementary Figure 5. Chromoplexy and templated insertions in MM. a-c) Three chromoplexy events observed in our series. Two events were acquired at relapse (PD26416e and PD26403d) and one was already present at SMM phase (PD26400a). d-h) Five out of 6 templated insertions are shown: in 4 out of the 5, *MYC* was specifically involved (PD26409a, PD26411a, PD26426e and PD26432c).

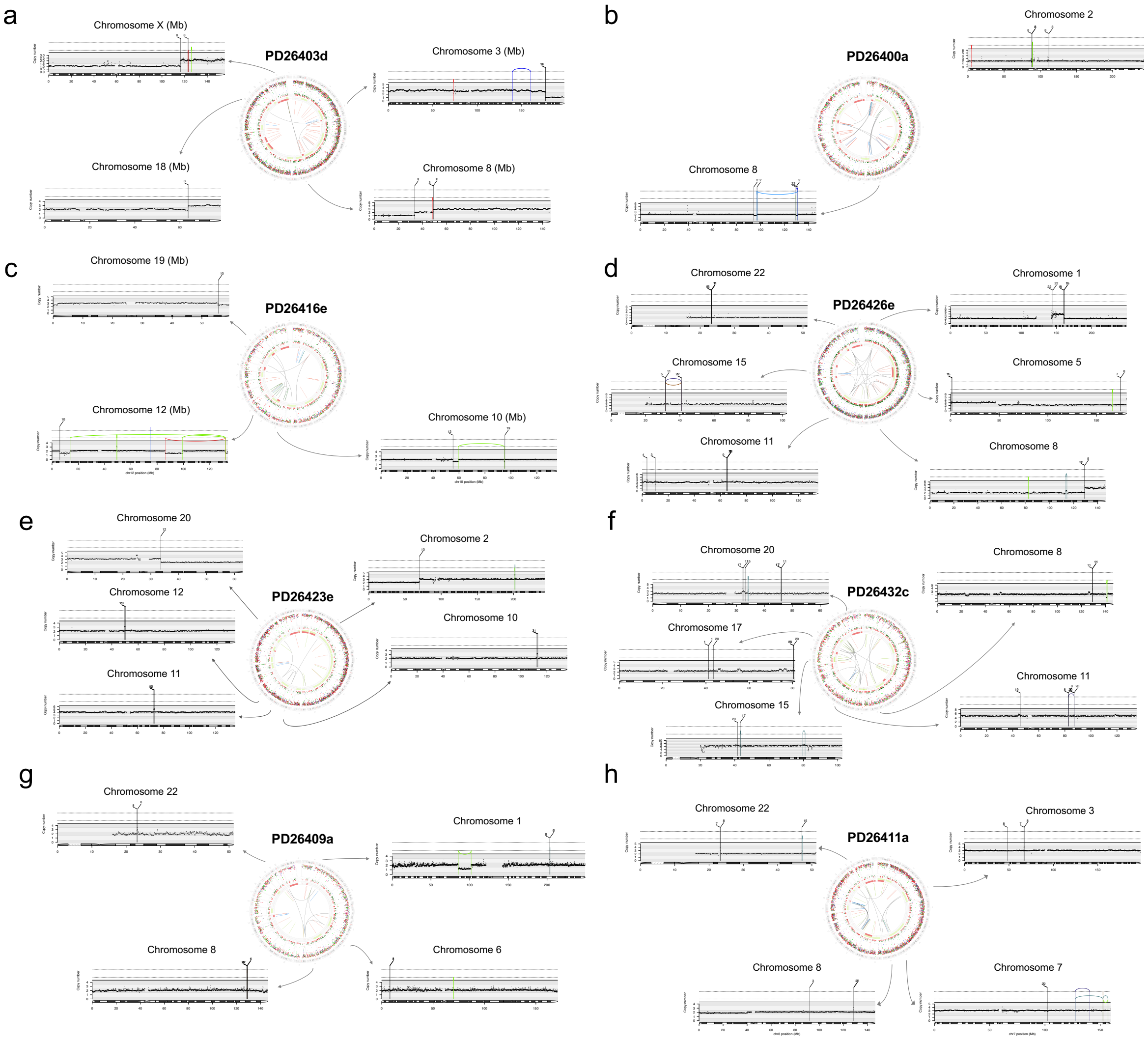

Supplementary Figure 6. SVs involving *CCND1*. a-g) Rearrangements/copy number plots for all *CCND1* translocations: the “simple” reciprocal (a, c, g, h and i) and the complex ones (b, d, e, f). The chromothripsis on PD26425 11q (c) was in close proximity but independent from the *CCND1*/*IGH* translocation. j) Map of the positions of *CCND1*/*IGH* translocation breakpoints on chromosome 14: note that they fall both on the IGH variable and the constant regions [66].

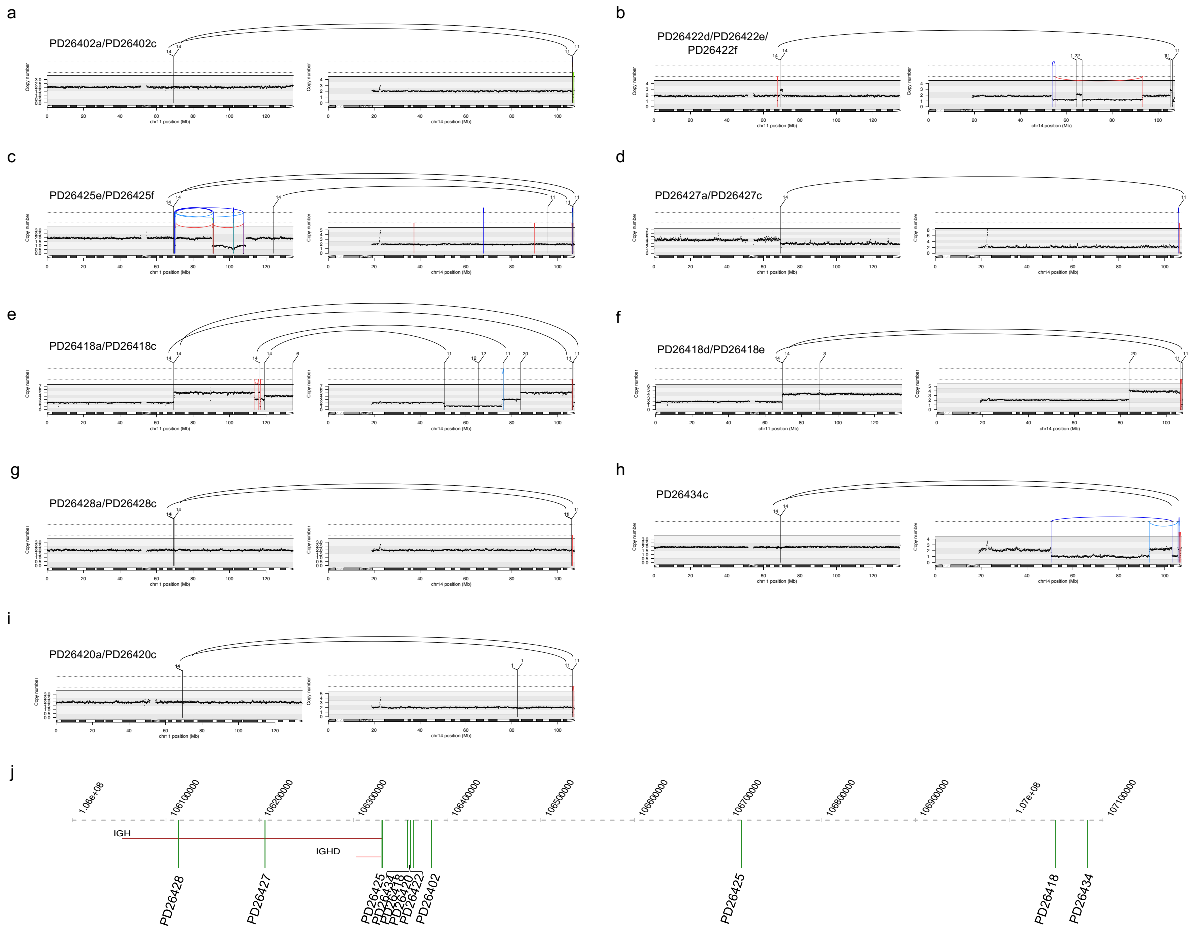

Supplementary Figure 7. MYC SVs and CNVs. Zoomed-in view of the chromosome 8q24 cytogenetic status extracted by battenberg in all samples that showed any SV involving *MYC.* Vertical black and blue lines correspond to translocations and inversions, respectively. All but two samples (PD26408 and PD26409) were characterized by the presence of focal deletions or gains. These changes may involve either the *MYC* locus or small regions upstream/downstream the oncogene. The H3K4me3 density is plotted at the bottom of each graph in red.

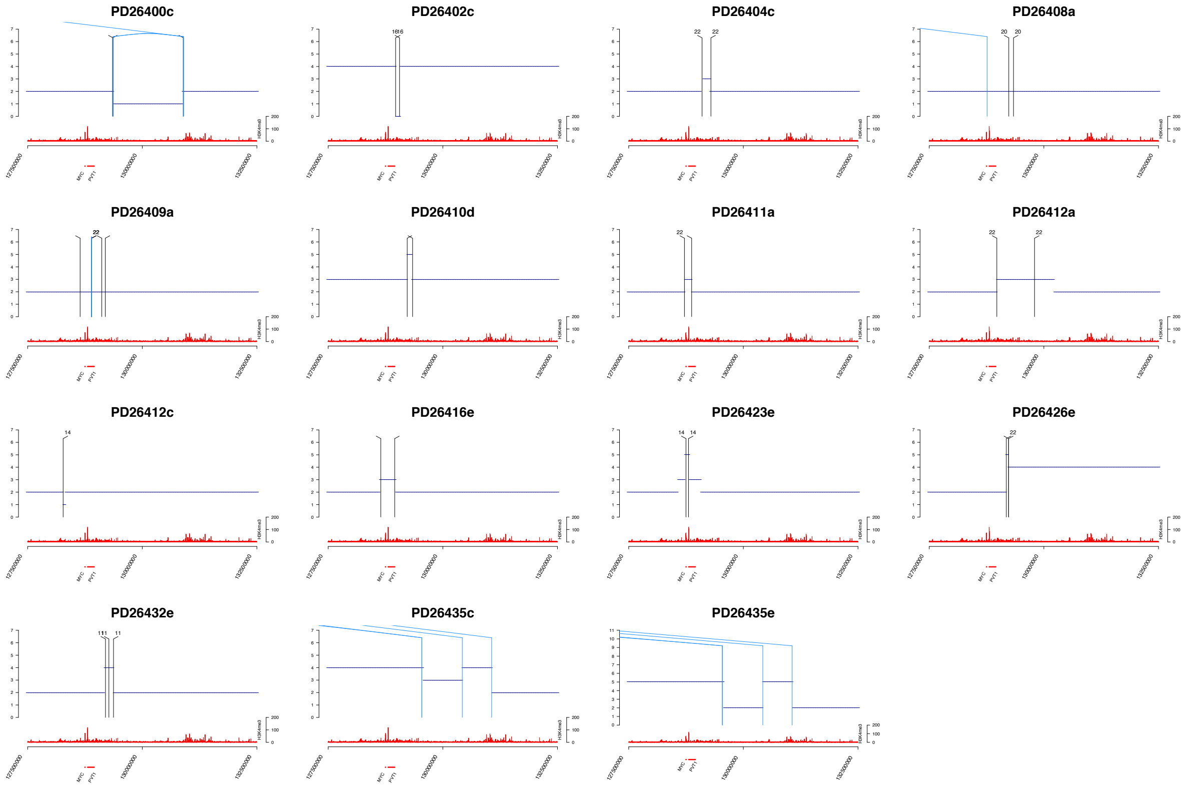

Supplementary Figure 8. SV convergent evolution. a) Example of convergent evolution driven by SVs. At relapse after XVI cycles of Elotuzumab-Lenalidomide-Dexamethasone, the clone with the translocation between *IGL* and *MYC* was replaced by another one characterized by a changed copy number change profile and by a novel translocation on *MYC*, involving the *IGH* locus this time. This pattern suggests the existence of 2 different clones in the same patients harboring 2 different *MYC* aberrations. b) The translocations' CCF as estimated by digital PCR (Methods).

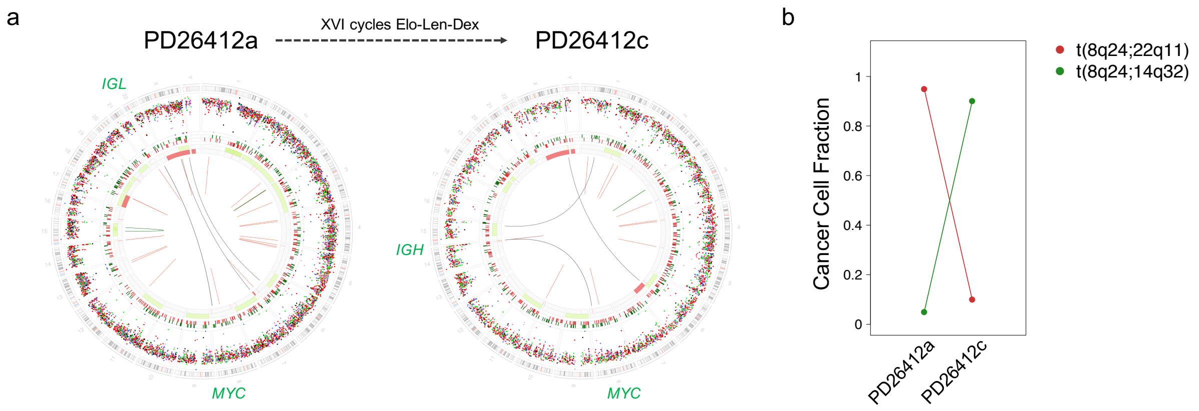

Supplementary Figure 9. Whole genome duplication in MM. a-c) The cytogenetic profile of 3 out of 4 MM relapsed patients that experienced a WGD. Among these, PD26414 showed evidence of a WGD subclone in the first sample already, which nevertheless underwent further amplification; for PD26423, only a relapsed-refractory sample was available; PD26435 was the only ones with a clear evidence of WGD acquisition at MM relapse. d) Example of chromothripsis occurred after relapse on a chromosomal region already involved by a complex event. The relapsed sample (PD26435e) also underwent a WGD which explains the increased ploidy.

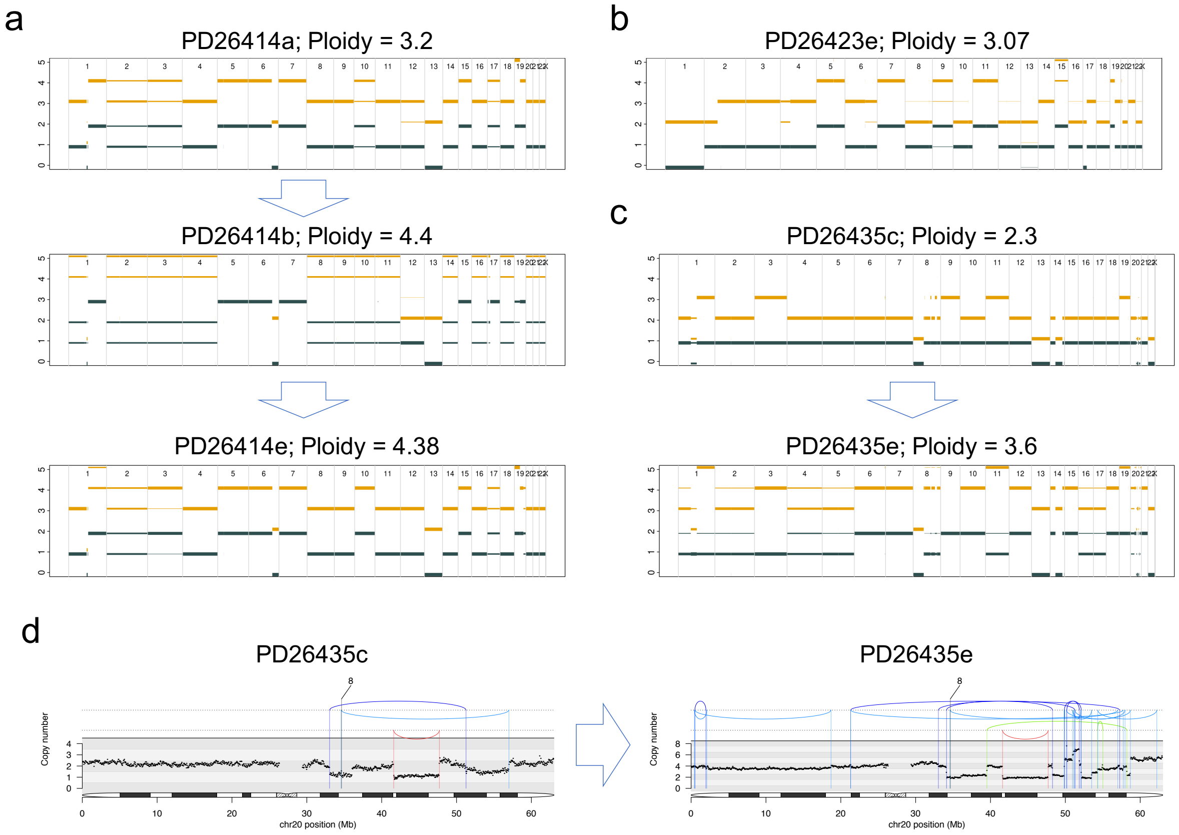

Supplementary Figure 10. Molecular time (MT) plots for all patients with more than one gain in the cohort. The MT of each chromosome gain (allele-specific copy number of 2:1) is plotted with a blue dot. The red and green dots represent the second (3:1) and the third extra gain (4:1) respectively. Different time windows between independent multi-gain events are separated by a dashed green line. Interval of confidences were generated using a bootstrapping function for each molecular time value.

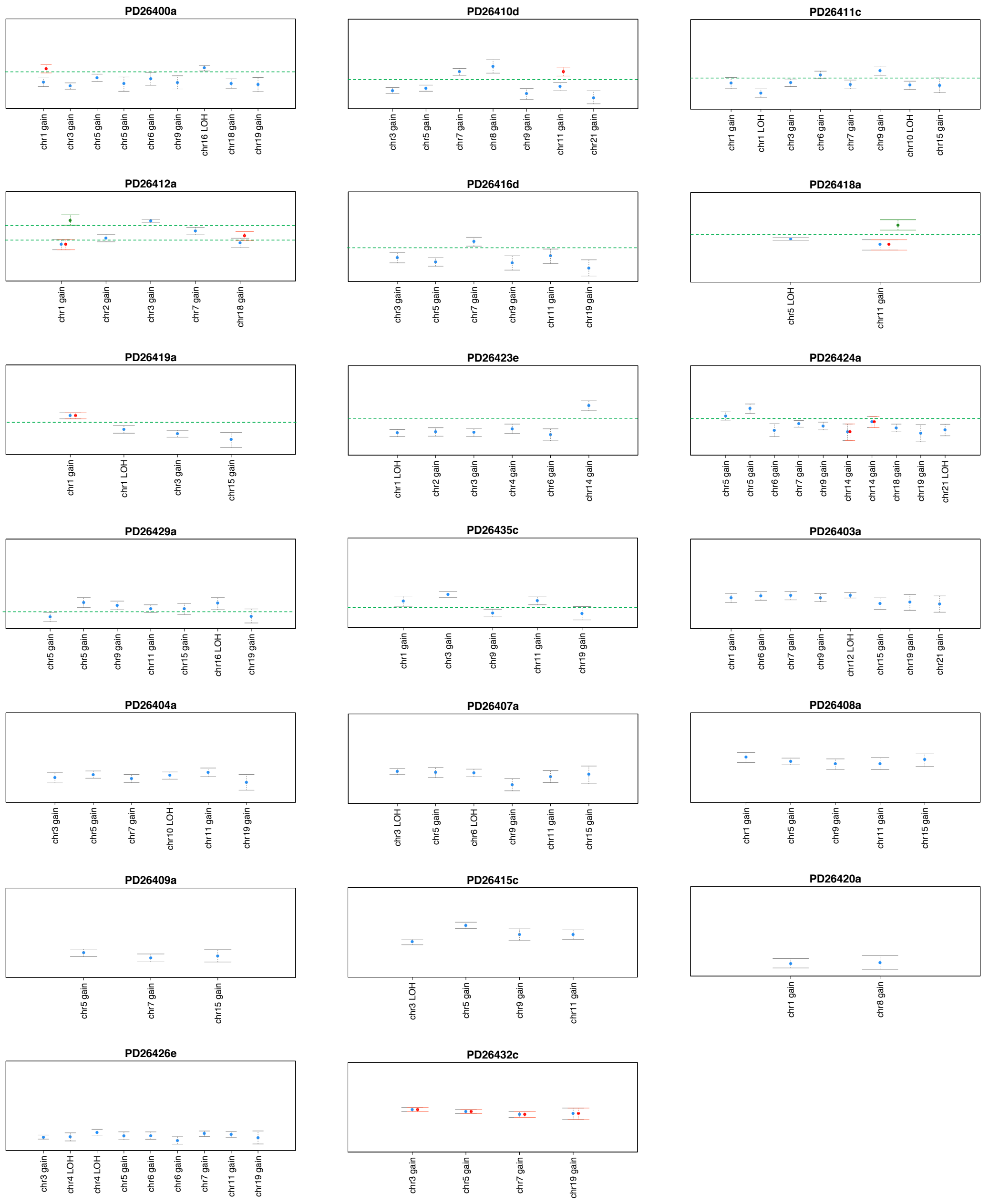

Supplementary Figure 11. Validation of the molecular time analysis in serial samples. Two examples where at least one clonal chromosomal gain was acquired or lost between samples (purple boxes). Unstable CNAs, while seemingly clonal in one sample, were clearly acquired or lost late in disease evolution, and consistently were assigned a late time window by molecular time analysis (right). Interval of confidences were generated using a bootstrapping function for each molecular time value.

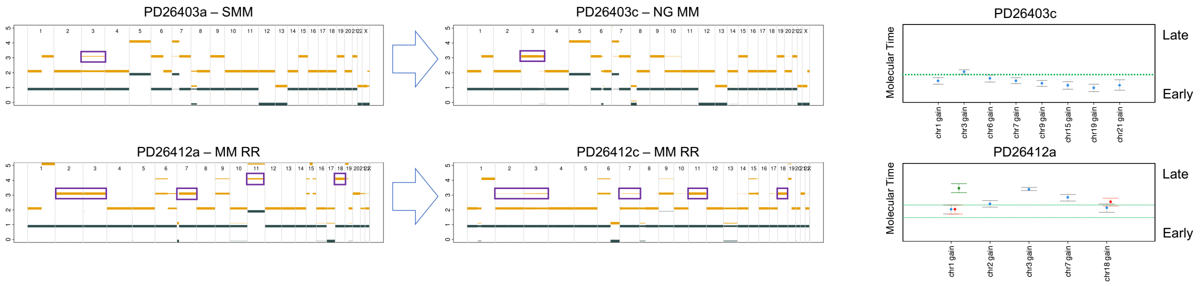

Supplementary Figure 12. Chronological reconstruction of driver events in MM. Two-dimensional density plots showing the Dirichlet process clustering of mutations by cancer cell fraction at each time point in 9 emblematic patients. On the x- and y axes are plotted mutations from 2 different samples collected at different time points from the same patients. Increasing intensity of red indicates the location of a high posterior probability of a cluster. The main SVs, CNAs and mutations were assigned to different cluster according to their cancer cell fraction and clonality over the time. Using this approach, the phylogenetic tree of each case was reconstructed and annotated with the main drivers as shown on the right of each plot.

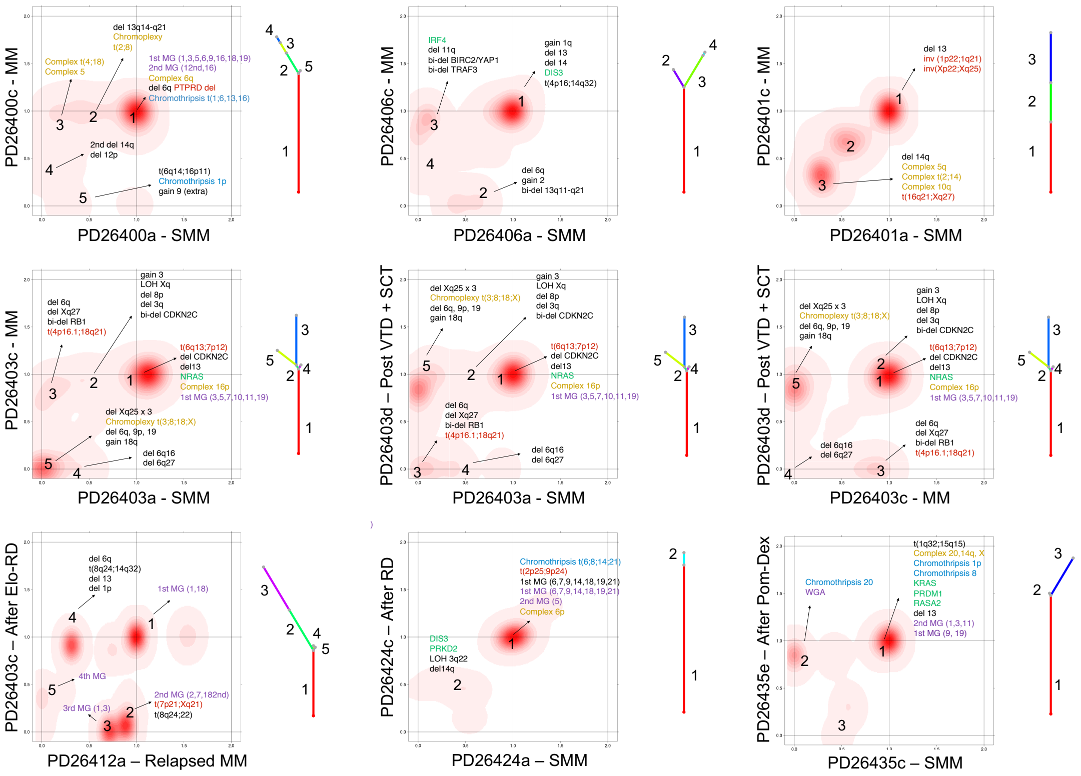

Supplementary Figure 13. Five examples of correlations between more than 2 MM driver events. a) a mutually exclusive pattern between t(11;14), t(14;16), del *TRAF3* and hyperdiploid; b) the selected gates pick up on the logically equivalent formulation that samples with del*TRAF3* co-occur either with non t(11;14) or with *NFKBIA* mutation; c) four way mutual exclusivity and co- occurrence between del*CYLD,* t(4;14), t(11;14) and hyperdiploid; d) the co-occurrence of *CDKN2C* and *FAM46c* deletions is mutually exclusive with t(4;14); e) TRAF3 deletion is associated with t(4;14) and del13q14.

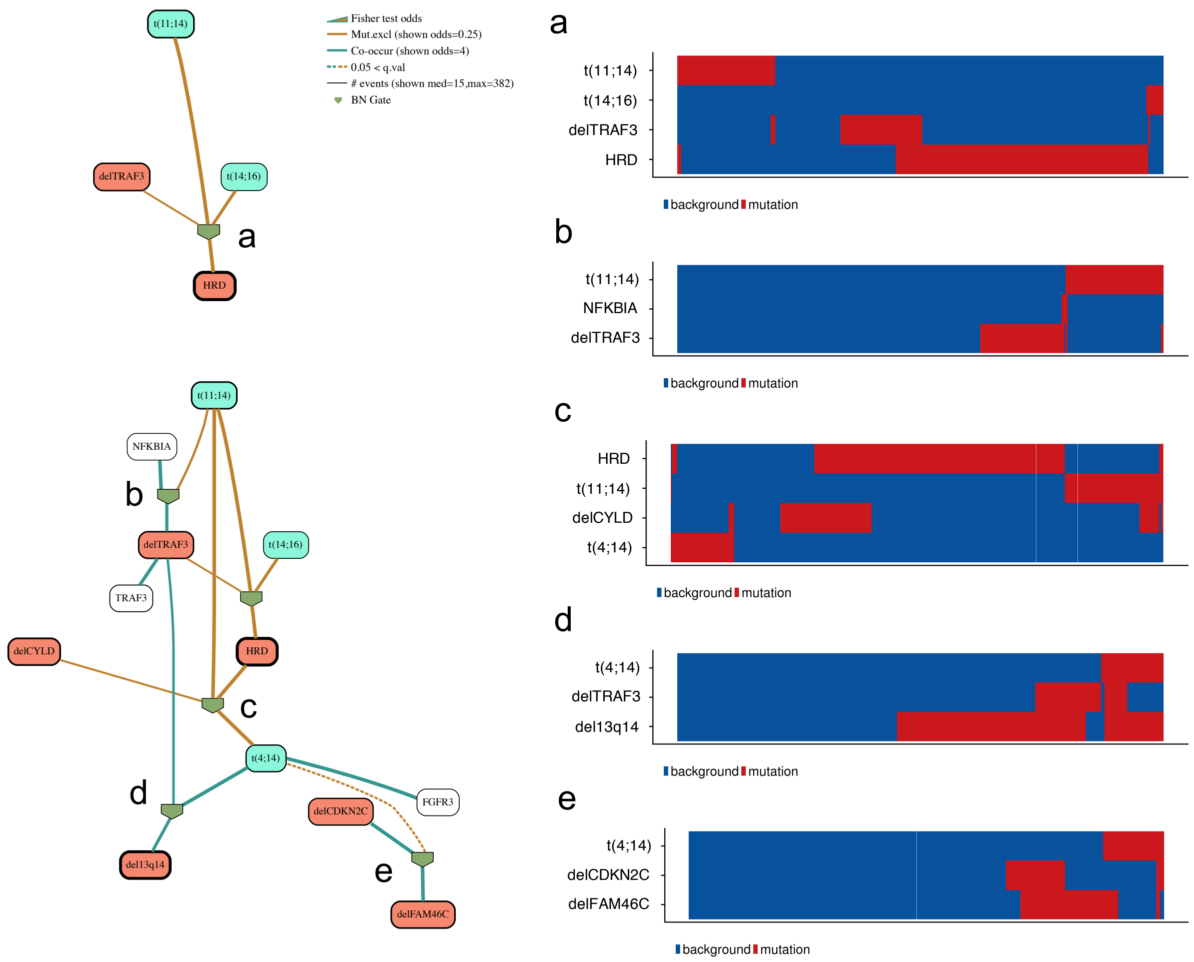

Supplementary Figure 14. *hdp* diagnostic plots showing the clustering stability and absence of strong trends over the posterior samples collected in 4 independent sampling chains. The dashed vertical red line represents the choice of burn-in time and number of iterations between collected samples

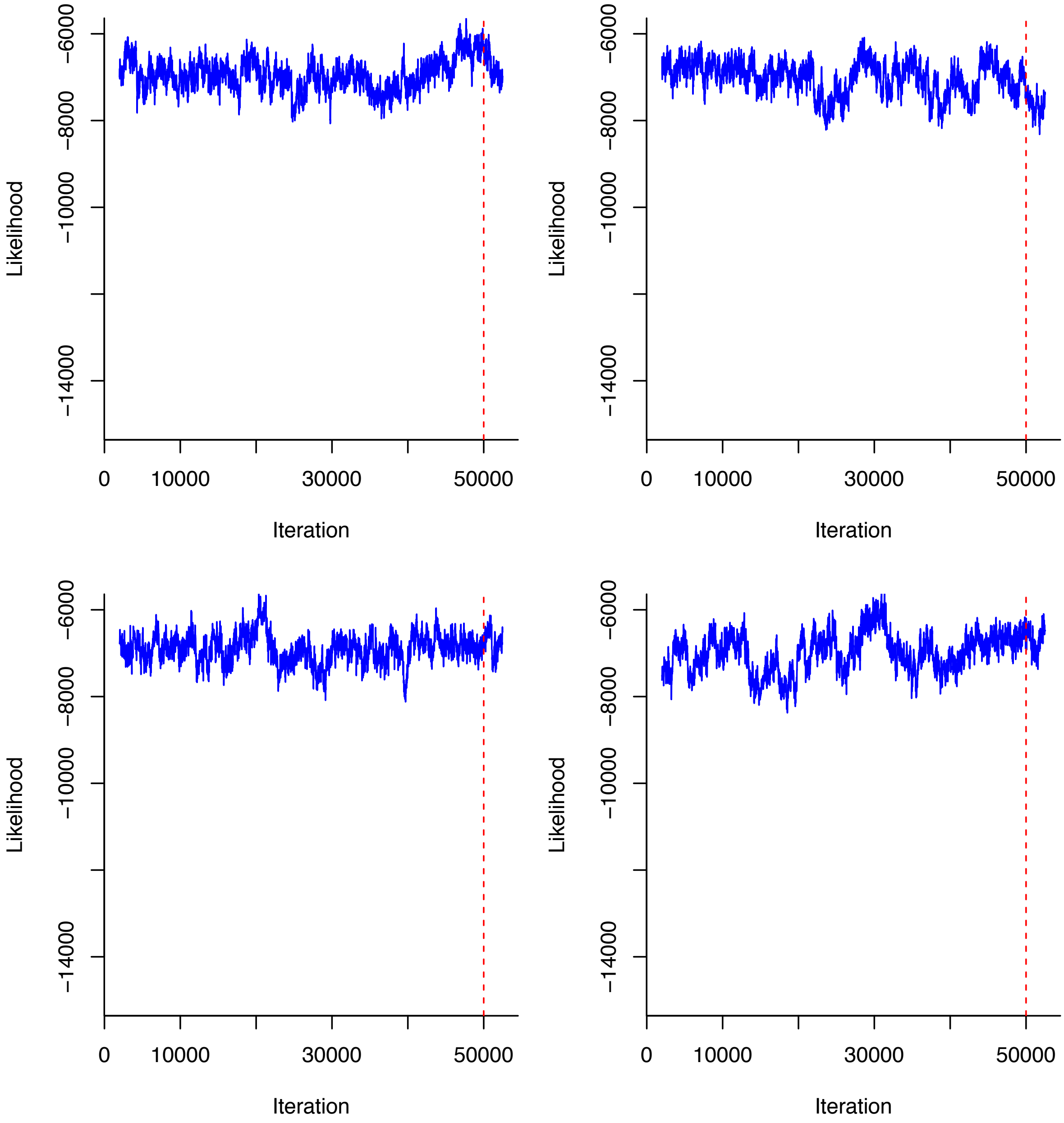

Supplementary Figure 15. Workflow to generate the final exome-based clustering.

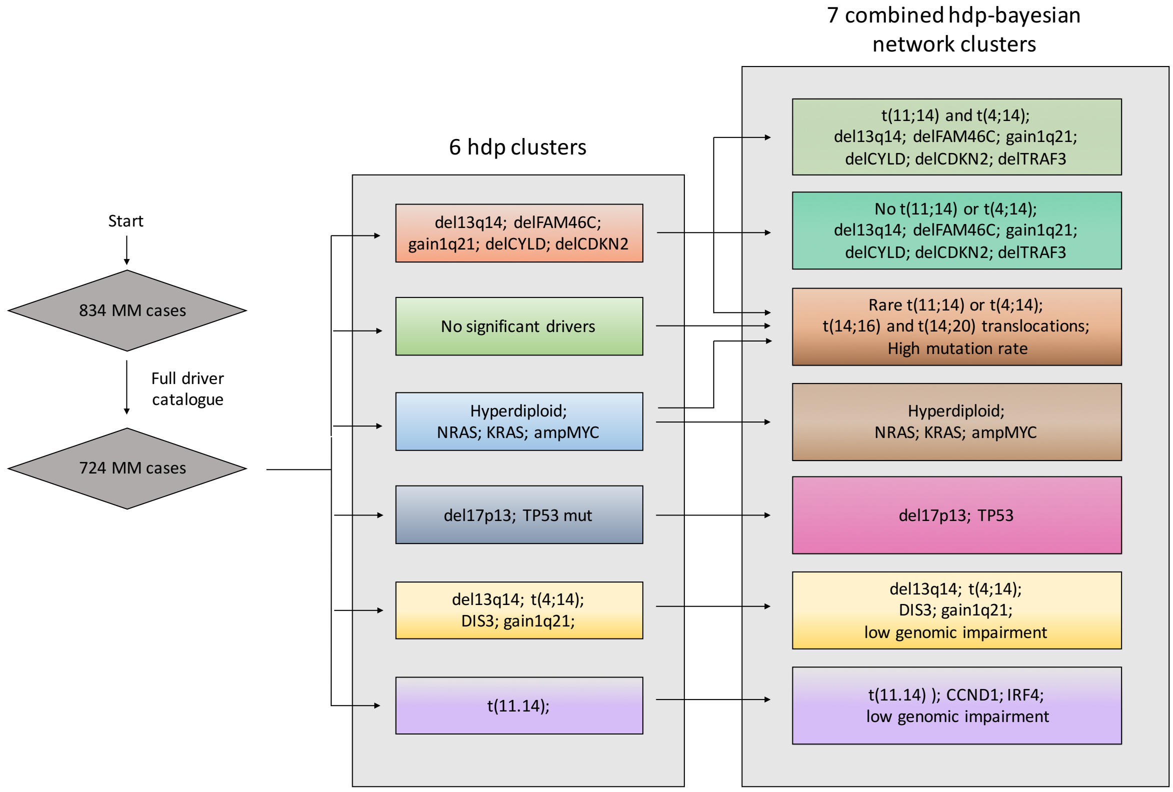

Supplementary Figure 16. Two examples of the phylogenetic three generation from the Dirichlet clustering data (2D-plot). In the first example (right) 2 possibile phylogenetic tree solutions were generated. Conversely, in the second case (left) only one solution was extracted.

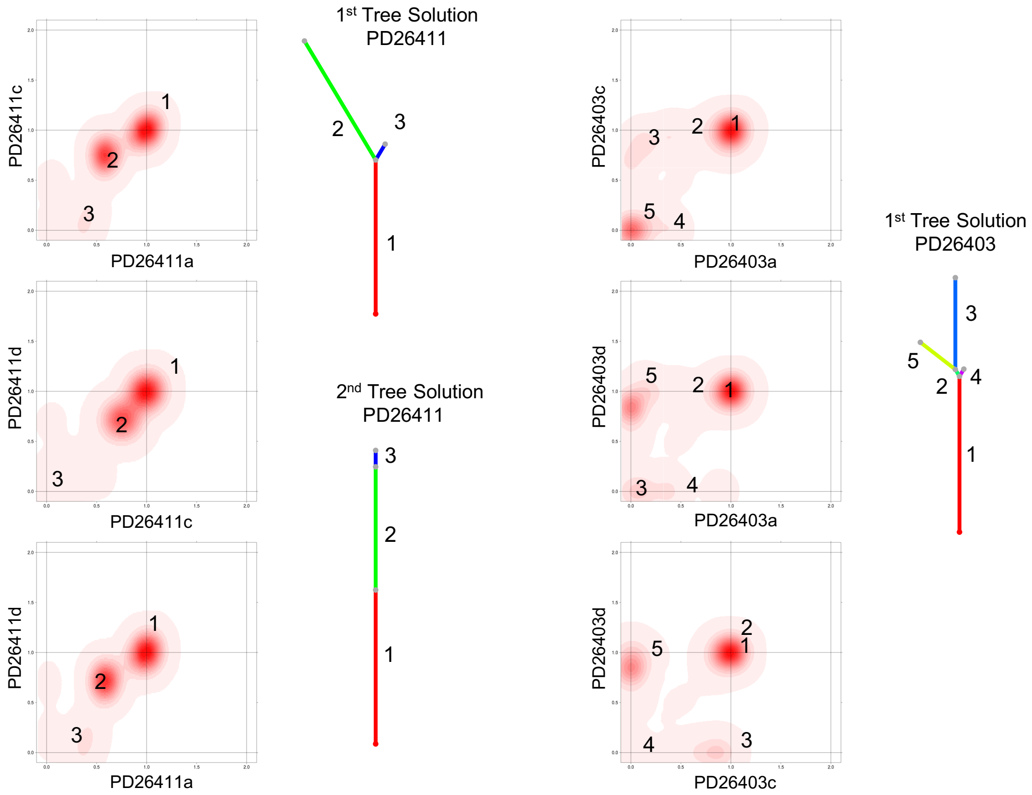

Supplementary Figure 17. Impact of large chromosomal duplications on SNVs VAF. a) Cartoon showing the rational of the molecular time approach. b-c) *mclust* was run on 2 chromosome gains dividing the SNV catalogue in 2 main groups: one where mutations were detected on 2 alleles (c-VAF ~66%) and one where mutations were observed only of one allele out of three (c-VAF ~33%). d) *mclust* was run on a copy neutral loss of heterozygosity (CN-LOH), and this explains why duplicated and non-duplicated mutations had 100% and 50% c-VAF respectively. e) *mclust* was also run in presence of 2 extra copies of the same chromosome, dividing mutations three groups: i) c-VAF ~75% (pre-gains); ii) c-VAF 50% (between the 2 gains); iii) c-VAF ~25% on one single allele out of four. The existence of 3 clusters, rather than of 2, suggests that the first and second chromosome 11 gains were acquired in 2 independent multi gain events.

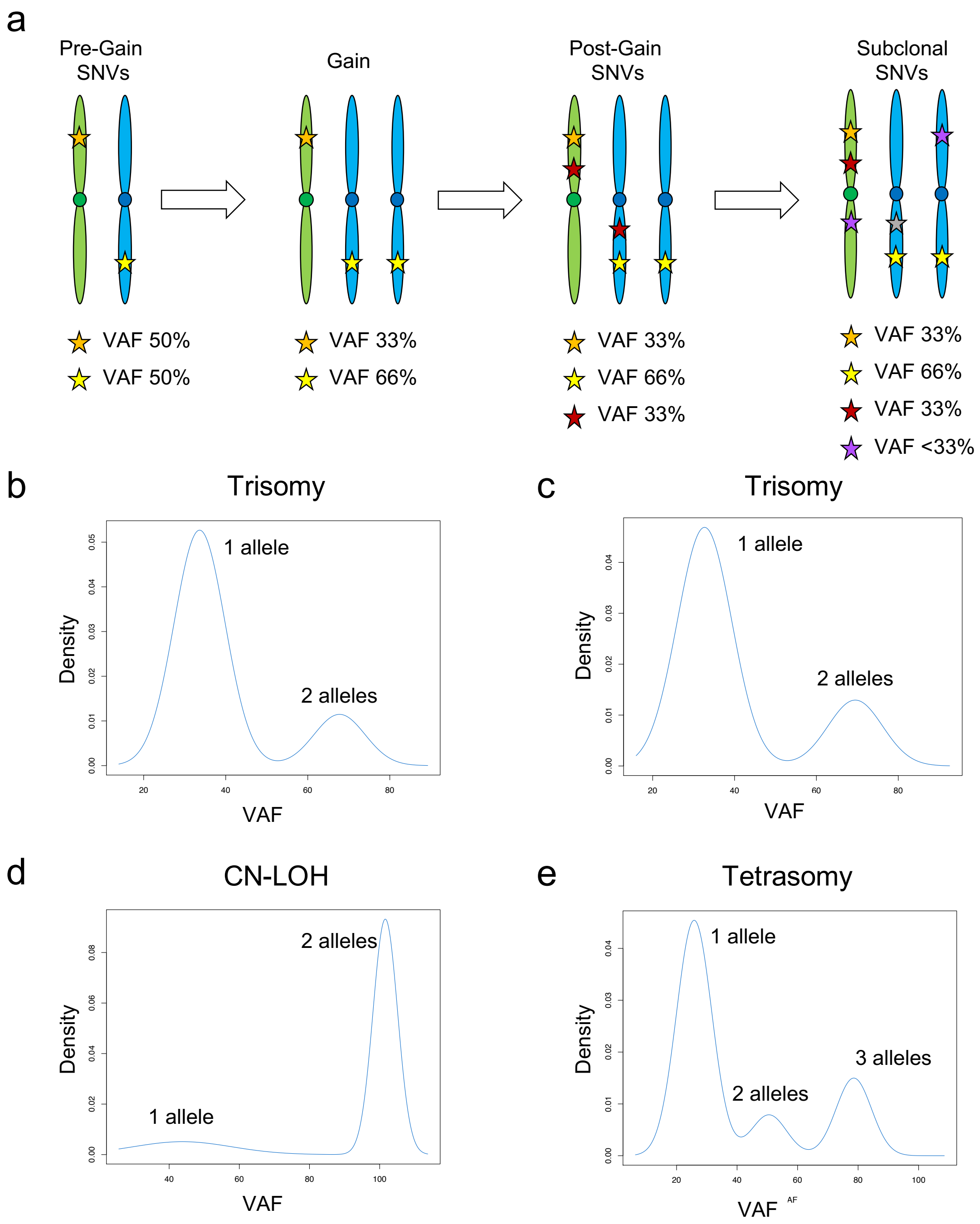
a
Pre-Gain SNVs
Gain
Post-Gain SNVs
Subclonal SNVs
VAF 50%
VAF 50%
VAF 33%
VAF 66%
VAF 33%
VAF 66%
VAF 33%
VAF 33%
VAF 66%
VAF 33%
VAF <33%
b
Trisomy
1 allele
2 alleles
Density
VAF
c
Trisomy
1 allele
2 alleles
Density
VAF
d
CN-LOH
2 alleles
1 allele
Density
VAF
e
Tetrasomy
1 allele
2 alleles
3 alleles
Density
VAF

Supplementary Figure 18. Example showing the “*mol_time*” function clustering part output (*mclust* based) for sample PD26410d.

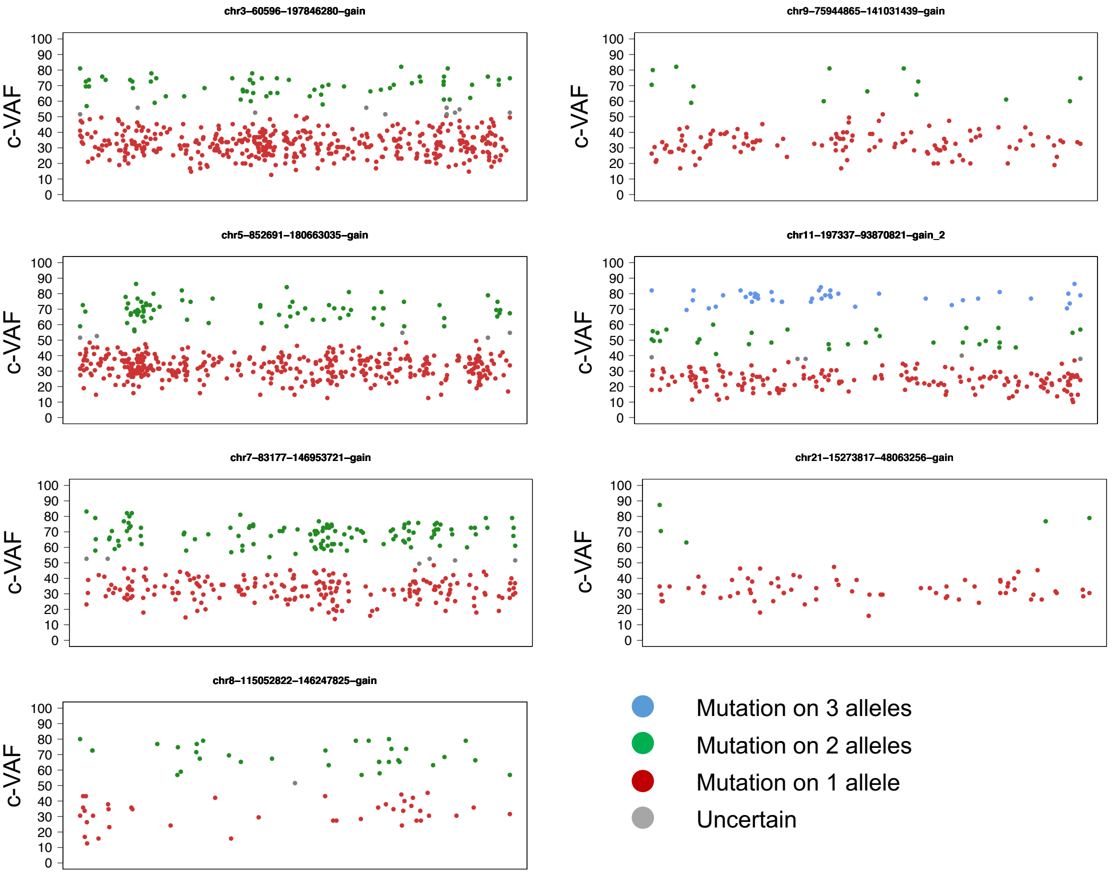

Supplementary Figure 19. Molecular time rationale. a) The burden of mutations duplicated (CN2) and therefore acquired before the chromosomal gain is proportional to the time in which the gain was acquired. b) High and low CN2 mutational burden reflect early and late chromosomal gain occurrence, respectively.

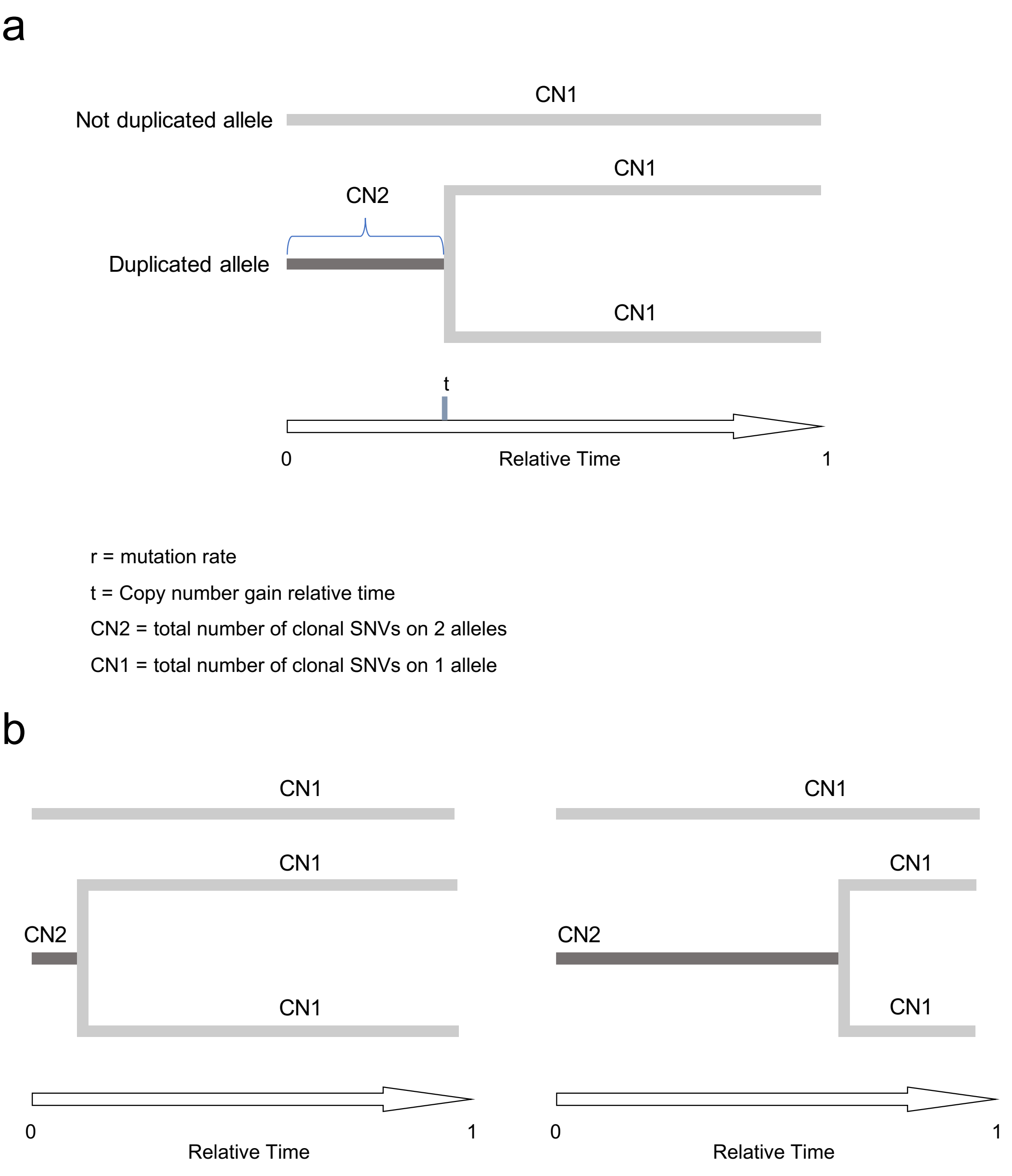

Supplementary Figure 20. Timing SVs' occurrence. a-b) Timing SVs' occurrence combining SV and copy number status. If occurring after a gain, any deletion on the not-duplicated allele will generate a LOH (2:0); and an involvement of one duplicated allele will generate a normal diploid segment (1:1). Conversely an involvement of the duplicated allele before the gain will generate a deletion (1:0). b) Example of a complex event supported by a t(3;22) translocation and multiple t(3;13) translocations causing a deletion after the chromosome duplication on a one of the duplicated alleles. In fact, SVs rearrangement-VAF is not duplicated and the CN status after this event is 2 (diploid). c-d) The c-VAF of SNP/SNV phased within the reads supporting the rearrangement reflects the relative time in which the SV was acquired.

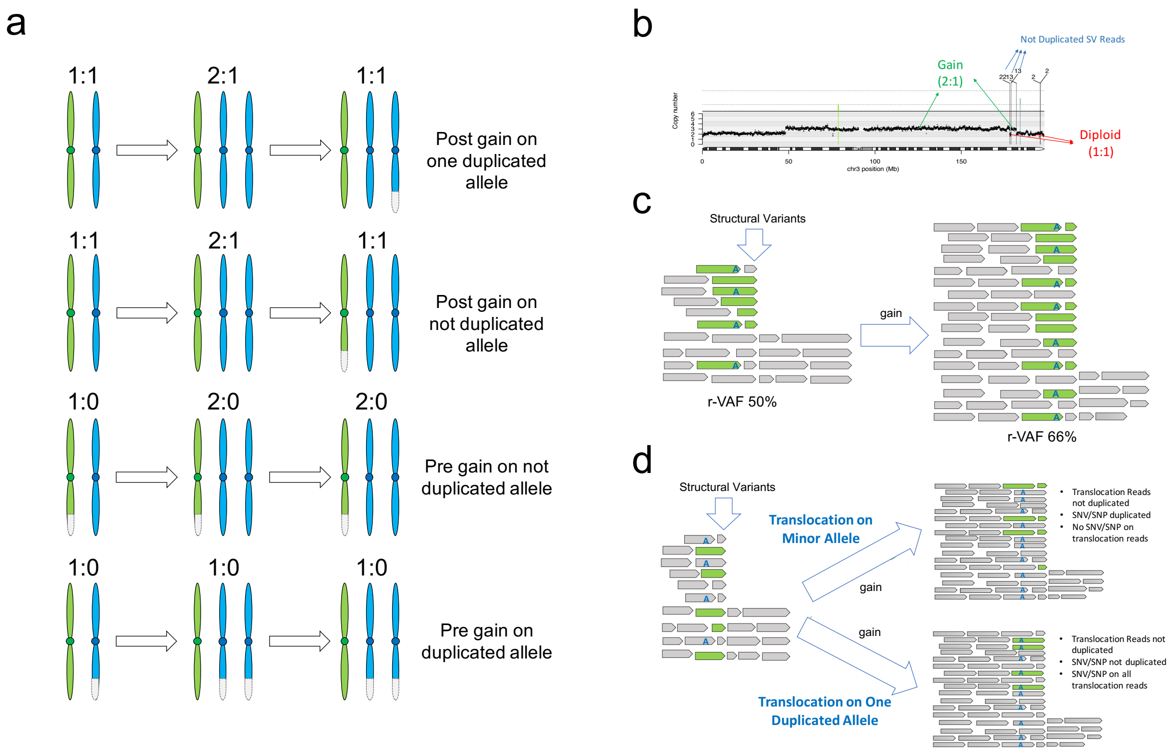

Supplementary Table 1. Digital PCR primers ordered from Sigma

ID	Sample ID	Sense Primer	Anti-sense Primer
**1**	PD26412a_8a	GAAGGTGTGGTTTAAAGAG	CAGAGATCAGGGATTTGA
**2**	PD26412a_8b	ATGCCATATTAAAATATGTATTC	GCTAGTGTTAACTTTGAG
**3**	PD26412c_8a	GTCCATAGGTTGTCTCTA	CCTTCATGACTTGATCAC
