# Supplementary material for: Genomic landscape and chronological reconstruction of driver events in multiple myeloma
Source: Nat Commun. 2019 Aug 23;10:3835. doi: 10.1038/s41467-019-11680-1 (PMC6707220; doi:10.1038/s41467-019-11680-1)
Supplement: Supplementary file 3 — Description of Additional Supplementary Files [file 41467_2019_11680_MOESM3_ESM.pdf]

Description of Additional Supplementary Files

File Name: Supplementary Data 1
Description: Sample characteristics and summary of the WGS.

File Name: Supplementary Data 2
Description: A summary of the treatments and collecting timing for each sample.

File Name: Supplementary Data 3
Description: Summary of dNdS driver discovery analysis and the concordance with the recently published Myeloma Genome Project study. The global p and q values for Myeloma Genome Project unique mutations were estimated performing the restricted hypothesis dNdS for these genes.

File Name: Supplementary Data 4
Description: Structure of the phylogenetic tree of each sample.

File Name: Supplementary Software 1
Description: This document contains the hierarchical dirichlet process (hdp) complete code used in the analysis. It is purely written in R. This report has been generated using the knitr R package.

File Name: Supplementary Software 2
Description: Tree finding algorithm. The code as written in LaTeX, using the algpseudocode package.
