# Supplementary material for: Genomic landscape and chronological reconstruction of driver events in multiple myeloma
Source: Nat Commun. 2019 Aug 23;10:3835. doi: 10.1038/s41467-019-11680-1 (PMC6707220; doi:10.1038/s41467-019-11680-1)
Supplement: Supplementary file 4 — Supplementary Data 1 [file 41467_2019_11680_MOESM4_ESM.pdf]

Supplementary Data 1

Sample ID	Coverage	Sample Type	Age	Sex	Phase	Ig Type	Isotype	Cytogenetic
**PD26400a**	38.74	tumour	69	M	SMM	IgG	Kappa	HRD
**PD26400b**	36.96	normal	-	-	-		-	-
**PD26400c**	38.54	tumour	72	M	DG	IgG	Kappa	HRD
**PD26401a**	45.53	tumour	74	M	SMM	IgG	Kappa	
**PD26401b**	36.52	normal	-	-	-		-	-
**PD26401c**	43.15	tumour	74	M	DG	IgG	Kappa	
**PD26402a**	43.4	tumour	55	F	SMM	IgG	Kappa	t(11;14)
**PD26402b**	33.75	normal	-	-	-	-	-	-
**PD26402c**	41.06	tumour	57	F	DG	IgG	Kappa	t(11;14)
**PD26403a**	39.32	tumour	57	F	SMM	IgG	Kappa	HRD
**PD26403b**	34.49	normal	-	-	-	-	-	-
**PD26403c**	40.06	tumour	57	F	DG	IgG	Kappa	HRD
**PD26403d**	39.07	tumour	59	F	RR	IgG	Kappa	HRD
**PD26404a**	41.19	tumour	64	M	SMM	IgG	Kappa	HRD
**PD26404b**	36.1	normal	-	-	-	-	-	-
**PD26404c**	36.12	tumour	65	M	DG	IgA	Kappa	HRD
**PD26405a**	42.65	tumour	64	F	SMM	IgA	Kappa	t(4;14)
**PD26405b**	35.5	normal	-	-	-	-	-	-
**PD26405c**	41.13	tumour	64		DG	IgA	Kappa	t(4;14)
**PD26406a**	35.27	tumour	61	M	SMM	IgG	Kappa	t(4;14)
**PD26406b**	32.15	normal	-	-	-	-	-	-
**PD26406c**	39.36	tumour	63	M	DG	IgG	Kappa	t(4;14)
**PD26407a**	36.09	tumour	49	M	SMM	IgG	Kappa	HRD
**PD26407b**	34.37	normal	-	-	-	-	-	-
**PD26407c**	43.82	tumour	49	M	DG	IgG	Kappa	HRD
**PD26408a**	47.86	tumour	53	M	SMM	IgG	Kappa	HRD
**PD26408b**	29.16	normal	-	-	-	-	-	-
**PD26408c**	46.73	tumour	53	M	DG	IgG	Kappa	HRD
**PD26409a**	41.47	tumour	64	F	MGUS	IgG	Kappa	HRD
**PD26409b**	34.57	normal	-	-	-	-	-	-
**PD26409c**	45.15	tumour	65	F	DG	IgG	Kappa	HRD
**PD26410b**	33.31	normal	-	-	-	-	-	-
**PD26410d**	37.7	tumour	66	M	RR	IgG	Lambda	HRD
**PD26411a**	39.26	tumour	65	M	RR	IgA	Lambda	HRD
**PD26411b**	34.96	normal	-	-	-	-	-	-
**PD26411c**	36.37	tumour	64	M	RR	IgA	Lambda	HRD
**PD26411d**	34.9	tumour	64	M	RR	IgA	Lambda	HRD
**PD26412a**	34.39	tumour	50	M	RR	IgG	Kappa	HRD
**PD26412b**	30.41	normal	-	-	-	-	-	-
**PD26412c**	36.92	tumour	54	M	RR	IgG	Kappa	HRD

**PD26412d**	36.13	tumour	54	M	RR	IgG	Kappa	HRD
**PD26414a**	35.18	tumour	54	F	RR	IgA	Kappa	t(4;14)
**PD26414b**	36.09	tumour	-	-	-	-	-	-
**PD26414d**	36.54	normal	55	F	RR	IgA	Kappa	t(4;14)
**PD26414e**	31.39	tumour	58	F		IgA	Kappa	t(4;14)
**PD26414f**	34.19	tumour	58	F	RR	IgA	Kappa	t(4;14)
**PD26414g**	31.69	tumour	58	F	RR	IgA	Kappa	t(4;14)
**PD26415b**	35.84	normal	-	-	-	-	-	-
**PD26415c**	38.41	tumour	55	M	RR	IgG	Kappa	HRD
**PD26415g**	39.39	tumour	62	M	RR	IgG	Kappa	HRD
**PD26416b**	35.73	normal	-	-	-	-	-	-
**PD26416d**	36.12	tumour	52	F	RR	IgG	Kappa	HRD
**PD26416e**	36.71	tumour	53	F	RR	IgG	Kappa	HRD
**PD26418a**	40.35	tumour	71	M	RR	IgA	Kappa	t(11;14)
**PD26418b**	32.84	normal	-	-	-	-	-	-
**PD26418c**	35.63	tumour	71	M	RR	IgA	Kappa	t(11;14)
**PD26418d**	42.96	tumour	72	M	RR	IgA	Kappa	t(11;14)
**PD26418e**	34.5	tumour	72	M	RR	IgA	Kappa	t(11;14)
**PD26419a**	36.31	tumour	65	F	DG	LC	Kappa	t(8q24.3;14)
**PD26419b**	37.4	normal	-	-	-	-	-	-
**PD26419c**	36.35	tumour	67	F	RR	LC	Kappa	t(8q24.3;14)
**PD26419d**	37.74	tumour	67	F	RR	LC	Kappa	t(8q24.3;14)
**PD26420a**	40.69	tumour	56	M	RR	IgM	Kappa	t(11;14)
**PD26420b**	34.24	normal	-	-	-	-	-	-
**PD26420c**	37.75	tumour	56	M	RR	IgM	Kappa	t(11;14)
**PD26422b**	35.22	normal	-	-	-	-	-	-
**PD26422d**	37.67	tumour	70	M	RR	IgA	Kappa	t(11;14)
**PD26422e**	37.33	tumour	70	M	RR	IgA	Kappa	t(11;14)
**PD26422f**	38.19	tumour	70	M	RR	IgA	Kappa	t(11;14)
**PD26423b**	37.57	normal	-	-	-	-	-	-
**PD26423e**	33.93	tumour	54	M	RR	IgG	Kappa	HRD
**PD26423g**	35.1	tumour	58	M	RR	IgG	Kappa	HRD
**PD26423h**	37.92	tumour	58	M	RR	IgG	Kappa	HRD
**PD26424a**	37.32	tumour	63	M	SMM	IgG	Kappa	HRD
**PD26424b**	36.12	normal	-	-	-	-	-	-
**PD26424c**	35.85	tumour	64	M	RR	IgG	Kappa	HRD
**PD26425b**	32.63	normal	-	-	-	-	-	-
**PD26425e**	36.54	tumour	63	M	RR	IgA	Kappa	t(11;14)
**PD26425f**	37.84	tumour	64	M	RR	IgA	Kappa	t(11;14)
**PD26426b**	33.18	normal	-	-	-	-	-	-
**PD26426e**	33.5	tumour	77	M	RR	IgG	Kappa	HRD
**PD26427a**	34.52	tumour	51	F	DG	LC	Lambda	t(11;14)
**PD26427b**	34.07	normal	-	-	-	-	-	-

**PD26427c**	35.71	tumour	52	F	RR	LC	Lambda	t(11;14)
**PD26428a**	29.63	tumour	41	M	DG	IgG	Kappa	t(11;14)
**PD26428b**	37.27	normal	-	-	-	-	-	-
**PD26428c**	37.67	tumour	42	M	RR	IgG	Kappa	t(11;14)
**PD26429a**	32.9	tumour	64	F	DG	IgG	Kappa	HRD
**PD26429b**	35.76	normal	-	-	-	-	-	-
**PD26432b**	36.71	normal	-	-	-	-	-	-
**PD26432c**	34.35	tumour	61	M	RR	IgA	Lambda	HRD
**PD26432e**	31.55	tumour	68	M	RR	IgA	Lambda	HRD
**PD26434b**	36.7	normal	-	-	-	-	-	-
**PD26434c**	34.46	tumour	58	M	DG	IgG	Kappa	t(11;14)
**PD26435b**	35.53	normal	-	-	-	-	-	-
**PD26435c**	38.05	tumour	65	M	RR	IgA	Kappa	HRD
**PD26435e**	37.38	tumour	67	M	RR	IgA	Kappa	HRD

HRD= hyperdiploid
SMM= smoldering multiple myeloma
DG= diagnosis
RR= relapse
