# Supplementary material for: Genomic landscape and chronological reconstruction of driver events in multiple myeloma
Source: Nat Commun. 2019 Aug 23;10:3835. doi: 10.1038/s41467-019-11680-1 (PMC6707220; doi:10.1038/s41467-019-11680-1)
Supplement: Supplementary file 5 — Supplementary Data 2 [file 41467_2019_11680_MOESM5_ESM.pdf]

Supplementary Data 2

Sample ID	Status	date_sampling	Previous Treatment	Previous SCT
**PD26400a**	SMM	19/08/2008	-	-
**PD26400c**	MM DG	12/01/2012	-	-
**PD26401a**	SMM	30/03/2010	-	-
**PD26401c**	MM DG	06/10/2010	-	-
**PD26402a**	SMM	20/04/2010	-	-
**PD26402c**	MM DG	20/02/2012	-	-
**PD26403a**	SMM	21/04/2010	-	-
**PD26403c**	MM DG	19/10/2010	-	-
**PD26403d**	MM RR	29/12/2011	After VTD + SCT	yes
**PD26404a**	SMM	09/09/2010	-	-
**PD26404c**	MM DG	23/02/2011	-	-
**PD26405a**	SMM	03/11/2010	-	-
**PD26405c**	MM DG	18/01/2011	-	-
**PD26406a**	SMM	18/01/2010	-	-
**PD26406c**	MM DG	17/01/2012	-	-
**PD26407a**	SMM	23/02/2011	-	-
**PD26407c**	MM DG	12/10/2011	-	-
**PD26408a**	SMM	26/10/2011	-	-
**PD26408c**	MM DG	26/12/2011	-	-
**PD26409a**	SMM	21/03/2012	-	-
**PD26409c**	MM DG	28/01/2014	-	-
**PD26410d**	MM RR	19/06/2008	After VRD	no
**PD26411c**	MM RR	18/10/2006	After VIII cycles Bortezomib + Tanespimycin	yes
**PD26411d**	MM RR	24/01/2007	After XII cycles Bortezomib + Tanespimycin	yes
**PD26411a**	MM RR	17/04/2007	After Bortezomib + Perifosine	yes
**PD26412a**	MM RR	12/02/2008	After Marizomib	yes
**PD26412c**	MM RR	19/10/2011	After Elotuzumab - RD	yes
**PD26412d**	MM RR	11/11/2011	After Elotuzumab - RD	yes
**PD26414a**	MM RR	06/03/2007	After VRD / CTD	no
**PD26414b**	MM RR	13/11/2008	After VCTD	no
**PD26414e**	MM RR	21/09/2010	After Vorinostat-RD	no
**PD26414f**	MM RR	07/12/2010	After Velcade-CXCR inhibitor 3 cycles	no
**PD26414g**	MM RR	07/03/2011	After MLP-Dex	no
**PD26415c**	MM RR	09/01/2006	After Tanespimycin	no
**PD26415g**	MM RR	11/04/2013	After Bortezomib + Tanespimycin	yes
**PD26416d**	MM RR	08/07/2008	After Bortezomib + Perifosine	no
**PD26416e**	MM RR	21/04/2009	After Bortezomib + Enzastaurin	no
**PD26418a**	MM RR	25/10/2007	After VIII VRD + I VRAD	no
**PD26418c**	MM RR	23/01/2008	After III VRAD and 1 Cytoxan	no
**PD26418d**	MM RR	06/04/2009	After HD MLP and VRD and Cytoxan	yes
**PD26418e**	MM RR	01/09/2009	After DCEP-Thalidomide-Bortezomib	yes
**PD26419a**	MM DG	14/03/2005	-	no
**PD26419c**	MM RR	30/07/2007	After TD and Bortezomib + Perifosine	no
**PD26419d**	MM RR	08/07/2008	After VCTD (12 cycles)	no
**PD26420a**	MM RR	01/01/2007	After TD/PAD and HD MPL/Dex maintenance	yes
**PD26420c**	MM RR	18/05/2007	After SGN40	yes
**PD26422d**	MM RR	11/10/2007	After 10 VRD/DCEP and 2nd HD MPL	yes
**PD26422e**	MM RR	24/05/2007	After 8 cycles Aplidin	yes
**PD26422f**	MM RR	08/04/2008	After 8 cycles Bortezomib + Perifosine	yes
**PD26423e**	MM RR	08/04/2008	After VD	yes
**PD26423g**	MM RR	29/06/2011	After VRD	yes
**PD26423h**	MM RR	14/12/2011	After Bortezomib/LY2127399	yes
**PD26424a**	SMM	21/10/2010	-	-
**PD26424c**	MM RR	20/10/2011	After RD	no
**PD26425e**	MM RR	01/07/2008	After Bortezomib + mTOR inhibitor	yes

**PD26425f**	MM RR	09/01/2009	After SGN40 + Lenalidomide	yes
**PD26426e**	MM RR	28/03/2013	After VRD and After anti-DKK1	no
**PD26427a**	MM DG	19/09/2006	-	-
**PD26427c**	MM RR	15/03/2007	After VRD	no
**PD26428a**	MM DG	08/01/2007	-	-
**PD26428c**	MM RR	17/06/2008	After VRD	no
**PD26429a**	MM DG	28/03/2007		-
**PD26432c**	MM RR	09/09/2008	After VRD	no
**PD26432e**	MM RR	02/12/2014	After Bortezomib Maintenance	no
**PD26434c**	MM DG	08/05/2014	-	-
**PD26435c**	MM RR	14/01/2008	After VD + HSP90 inhibitor	no
**PD26435e**	MM RR	16/06/2010	After PomDex	no

VRD = Bortezomib -Lenalidomide-Dexamethasone
PomDex = Pomalidomide – Dexamethasone
VD = Bortezomib -Dexamethasone
MPL = Melphalan
HD = High dose
CTD = Cyclophosphamide-Thalidomide-Dexamethasone
