# Supplementary material for: Genomic landscape and chronological reconstruction of driver events in multiple myeloma
Source: Nat Commun. 2019 Aug 23;10:3835. doi: 10.1038/s41467-019-11680-1 (PMC6707220; doi:10.1038/s41467-019-11680-1)
Supplement: Supplementary file 6 — Supplementary Data 3 [file 41467_2019_11680_MOESM6_ESM.pdf]

Supplementary Data 3

Gene Name	N. of Synonymous	N. of Missense	N. of Nonsense	N. of Ess. Splicing	N. of I Indels	pglobal_cv	qglobal_cv	MGP/Maura et al
MAX	0	21	6	2	0	0	0	shared
TP53	1	38	2	3	3	0	0	shared
IGLL5	88	149	1	8	8	0	0	Maura et al.
FAM46C	4	39	3	0	43	0	0	shared
DIS3	1	85	0	4	0	0	0	shared
BRAF	0	62	0	0	0	0	0	shared
KRAS	1	220	0	0	1	0	0	shared
NRAS	1	195	0	0	0	0	0	shared
TRAF3	0	30	20	2	19	0	0	shared
DUSP2	17	30	4	7	3	0	0	shared
TCL1A	5	18	0	1	1	4.12E-13	7.52E-10	Maura et al.
TRAF2	0	8	8	1	1	8.92E-13	1.49E-09	shared
CYLD	0	7	8	2	5	1.13E-12	1.75E-09	shared
LTB	11	19	0	9	0	2.08E-12	2.99E-09	shared
HIST1H1E	3	32	0	0	1	2.40E-12	3.22E-09	shared
BCL7A	6	6	0	8	2	2.74E-12	3.44E-09	Maura et al.
SP140	2	10	6	3	7	4.36E-12	5.15E-09	shared
NFKBIA	0	3	6	0	6	4.03E-11	4.50E-08	shared
EGR1	14	34	0	0	4	7.02E-11	7.42E-08	shared
PABPC1	6	28	0	0	2	8.72E-10	8.76E-07	Maura et al.
PRKD2	0	25	0	0	1	1.11E-09	1.03E-06	shared
TBC1D29	2	14	0	0	0	1.13E-09	1.03E-06	Maura et al.
IRF4	1	21	0	0	0	2.37E-09	2.07E-06	shared
RB1	0	3	6	2	4	2.66E-09	2.23E-06	shared
TGDS	0	10	1	1	2	5.48E-08	4.40E-05	shared
PTPN11	0	19	0	0	0	1.28E-07	9.90E-05	shared
FUBP1	1	7	2	5	2	1.54E-07	0.0001143	shared
RPL5	0	8	0	1	4	2.33E-07	0.00016701	Maura et al.
FGFR3	4	20	0	0	3	2.71E-07	0.00018786	shared
SAMHD1	0	16	2	2	0	7.31E-07	0.00048971	shared
ACTG1	7	27	2	0	1	1.10E-06	0.00071176	shared
HIST1H1B	3	11	0	0	3	1.37E-06	0.00086175	Maura et al.
NFKB2	5	21	4	0	2	1.44E-06	0.00087728	shared
KMT2B	3	12	8	1	2	1.57E-06	0.00092736	shared
KLHL6	3	18	1	3	0	2.90E-06	0.00166635	shared
RASA2	0	5	3	1	4	3.44E-06	0.00192214	shared
PIM1	4	18	1	0	0	6.72E-06	0.00364802	Maura et al.
PRDM1	3	11	3	0	3	2.52E-05	0.01282476	shared

**DTX1**	4	16	0	0	2	2.53E-05	0.01282476	Maura et al.
**SETD2**	5	14	6	1	5	2.55E-05	0.01282476	shared
**BHLHE41**	6	10	0	0	5	3.48E-05	0.01704383	Maura et al.
**RPL10**	2	13	0	0	0	4.18E-05	0.01997567	Maura et al.
**BTG1**	7	14	2	1	0	4.65E-05	0.02170359	Maura et al.
**RPS3A**	0	11	0	0	0	4.84E-05	0.02211069	Maura et al.
**CCND1**	16	21	0	0	0	6.42E-05	0.02866307	shared
**RPRD1B**	0	8	0	1	1	6.72E-05	0.02881599	Maura et al.
**HIST1H1D**	4	14	0	0	1	6.74E-05	0.02881599	Maura et al.
**ZNF292**	2	11	6	0	4	8.55E-05	0.03577625	shared
**RFTN1**	6	12	2	3	0	0.00012626	0.05176643	shared
**CDKN1B**	0	6	2	0	2	0.00014068	0.05630987	shared
**LCE1D**	0	3	0	0	1	0.00014295	0.05630987	Maura et al.
**XBP1**	3	9	0	0	3	0.00018562	0.07171385	shared
**IRF1**	0	6	3	0	0	0.00024666	0.09349807	Maura et al.
**POT1**	0	11	0	1	1	0.00027156	0.10102888	Maura et al.
**HIST1H2BK**	4	9	0	0	1	0.0002821	0.10304184	Maura et al.
**ABCF1***	41	2	1	0	0	1.01E-12	2.63E-11	MGP[#]
**ZFP36L1***	2	3	0	3	1	0.00191357	0.02309607	MGP
**TET2***	2	6	4	0	4	0.00315459	0.02309607	MGP
**ARID2***	2	2	3	1	2	0.00448743	0.02309607	MGP
**KDM6A***	2	0	2	0	1	0.00468623	0.02309607	MGP
**EP300***	2	6	4	2	2	0.00532986	0.02309607	MGP
**ARID1A***	1	7	1	1	4	0.03959958	0.14708415	MGP
**NCOR1***	2	5	3	1	1	0.07254475	0.23577044	MGP
**HUWE1***	3	25	0	6	2	0.08773504	0.25345677	MGP
**CDKN2C***	0	2	0	0	1	0.14062115	0.33767804	MGP
**SF3B1***	1	14	0	0	0	0.15084251	0.33767804	MGP
**ATM***	5	27	2	2	2	0.16358412	0.33767804	MGP
**NF1***	3	10	1	2	3	0.16883902	0.33767804	MGP
**CREBBP***	3	7	2	1	2	0.18374221	0.34123554	MGP
**DNMT3A***	0	6	2	0	0	0.37257116	0.58225971	MGP
**MAFB***	1	6	0	0	0	0.37693422	0.58225971	MGP
**MAF***	3	11	0	0	0	0.38890693	0.58225971	MGP
**KDM5C***	0	5	2	0	1	0.41122261	0.58225971	MGP
**UBR5***	2	12	3	1	1	0.42549748	0.58225971	MGP
**PIK3CA***	1	2	0	0	0	0.49231039	0.64000351	MGP
**IDH1***	0	4	0	0	0	0.7066186	0.85499735	MGP
**MAML2***	3	5	1	0	1	0.7234593	0.85499735	MGP
**MAN2C1**	1	6	0	0	0	0.84256609	0.95246602	MGP
**IDH2**	1	3	0	0	0	0.94331432	0.99975856	MGP
**KMT2C**	5	23	1	2	0	0.98913621	0.99975856	MGP

**ATRX**	0	12	1	0	0	0.99975856	0.99975856	MGP

*dNdS estimated using restricted multiple hypothesis for the mutations extracted only by MPG: *HUWE1*, *ATM*, *UBR5*, *KMT2C*, *ARID1A*, *CREBBP*, *ATRX*, *NF1*, *EP300*, *TET2*, *DNMT3A*, *KDM5C*, *SF3B1*, *KDM6A*, *NCOR1*, *ARID2*, *MAF*, *MAN2C1*, *ABCF1*, *MAML2*, *CDKN2C*, *MAFB*, *ZFP36L1*, *IDH1*, *PIK3CA*, *IDH2*

[#]MGP = Myeloma Genome Project
