# Supplementary material for: Genomic landscape and chronological reconstruction of driver events in multiple myeloma
Source: Nat Commun. 2019 Aug 23;10:3835. doi: 10.1038/s41467-019-11680-1 (PMC6707220; doi:10.1038/s41467-019-11680-1)

HDP classification 1252 MM

This document contains the hierarchical dirichlet process (hdp) complete code used in the analysis. It is purely written in R. This report has been generated using the knitr R package

Francesco Maura (fm6@sanger.ac.uk)

Daniel Leongamornlert (dl8@sanger.ac.uk)

Peter Campbell (pc8@sanger.ac.uk)

02/07/2019

Libraries

```
library(hdp)

## Run citation('hdp') for citation instructions,
##     and file.show(system.file('LICENSE', package='hdp')) for license detai
ls.

library(pheatmap)
library(survival)
library(hdp)
library(Matrix)
library(aCRM)

## aCRM 0.1.1

## Type aCRMNews() to see the change log

library(RColorBrewer)

#### reorder function for genetic interaction figure
reorder <- function(M, o){
  u <- M
  u[lower.tri(u)] <- t(M)[lower.tri(M)]
  u <- u[o,o]
  l <- M
  l[upper.tri(u)] <- t(M)[upper.tri(M)]
  l <- l[o,o]
  R <- u
  R[lower.tri(R)] <- l[lower.tri(R)]
  return(R)
}
```

```
rotatedLabel <- function(x0 = seq_along(labels), y0 = rep(par("usr")[3], leng
th(labels)), labels, pos = 1, cex=1, srt=45, ...) {
  w <- strwidth(labels, units="user", cex=cex)
  h <- strheight(labels, units="user",cex=cex)
  u <- par('usr')
  p <- par('plt')
  f <- par("fin")
  xpd <- par("xpd")
  par(xpd=NA)
  text(x=x0 + ifelse(pos==1, -1,1) * w/2*cos(srt/360*2*base::pi), y = y0 + if
else(pos==1, -1,1) * w/2 *sin(srt/360*2*base::pi) * (u[4]-u[3])/(u[2]-u[1]) /
(p[4]-p[3]) * (p[2]-p[1])* f[1]/f[2] , labels, las=2, cex=cex, pos=pos, adj=1
, srt=srt,...)
  par(xpd=xpd)
}
```

Preparation of data

```
############## upload matrix generated from COMMPASS AI9 version

all<-  read.delim("hdp_commpass_wgs_samples.txt", sep="\t", stringsAsFactors
= F, header=T)
all[all>0]<-1
genomicData<-sapply(data.frame(all),as.numeric)
rownames(genomicData)<- rownames(all)
mut_count<- genomicData
head(mut_count)
```

```
##             HDR t.11.14. t.4.14. t.14.16. t.14.20. ampMYC del13q14 del17p13
## MMRF_1032   0        0       0        0        0      0        0        0
## MMRF_1045   0        0       0        0        0      0        0        0
## MMRF_1169   0        0       0        0        0      0        1        1
## MMRF_1185   0        0       0        0        0      0        1        0
## MMRF_1270   0        0       0        0        0      0        1        0
## MMRF_1327   0        0       0        0        0      1        1        0
##             delCDKN2C delCYLD delFAM46C delTRAF2 delTRAF3 gain1q21 KRAS NRAS
## MMRF_1032           0       0         0        0        0        0    1    0
## MMRF_1045           0       0         0        0        0        1    0    0
## MMRF_1169           1       1         0        0        0        0    0    0
## MMRF_1185           1       0         1        0        1        0    0    0
## MMRF_1270           0       1         0        0        1        0    0    0
## MMRF_1327           1       1         1        0        1        1    0    0
##             IGLL5 DIS3 BRAF TRAF3 TP53 FAM46C DUSP2 ACTG1 HIST1H1E KLHL6
## MMRF_1032       0    0    0     0    0      0     0     0        0     0
## MMRF_1045       0    0    0     0    0      0     0     0        0     0
## MMRF_1169       0    0    0     0    0      0     0     0        0     0
## MMRF_1185       0    0    0     0    0      0     0     0        0     0
## MMRF_1270       0    0    0     0    0      0     0     0        0     0
## MMRF_1327       0    0    0     0    0      0     0     0        0     0
##             CYLD CCND1 IRF4 PABPC1 PIM1 TCL1A FGFR3 SP140 PRDM1 SETD2 TRAF2
```

```
## MMRF_1032    0     0    0      0    0     0     0     0     0     0     0
## MMRF_1045    0     0    0      0    0     0     0     0     0     0     0
## MMRF_1169    0     0    0      0    0     0     0     0     0     0     0
## MMRF_1185    0     0    0      0    0     0     0     0     0     0     0
## MMRF_1270    0     0    0      0    0     0     0     0     0     0     0
## MMRF_1327    0     0    0      0    0     0     0     0     0     0     0
##           NFKB2 RB1 BTG1 RFTN1 TBC1D29 HIST1H1B RASA2 DTX1 HIST1H2BK
## MMRF_1032     0   0    0     0       0        0     0    0         0
## MMRF_1045     0   0    0     0       0        0     0    0         0
## MMRF_1169     0   0    0     0       0        0     0    0         0
## MMRF_1185     0   0    0     0       0        0     0    0         0
## MMRF_1270     0   0    0     0       0        0     0    0         0
## MMRF_1327     0   0    0     0       0        0     0    0         0
##           HIST1H1D BCL7A FUBP1 CDKN1B XBP1 RPL5 LCE1D RPRD1B BHLHE41 POT1
## MMRF_1032        0     0     0      0    0    0     0      0       0    0
## MMRF_1045        0     1     0      0    0    0     0      0       0    0
## MMRF_1169        0     0     0      0    0    0     0      0       0    0
## MMRF_1185        0     0     0      0    0    0     0      0       0    0
## MMRF_1270        0     0     0      0    0    0     0      0       0    0
## MMRF_1327        0     0     0      0    0    0     0      0       0    0
##           RPS3A IRF1 TGDS RPL10 ZNF292 PTPN11 NFKBIA SAMHD1 KMT2B LTB
## MMRF_1032     0    0    0     0      0      0      0      0     0   0
## MMRF_1045     0    0    0     0      0      0      0      0     0   0
## MMRF_1169     0    0    0     0      0      0      0      0     0   0
## MMRF_1185     0    0    0     0      0      0      0      0     0   0
## MMRF_1270     0    0    0     0      0      0      0      0     0   0
## MMRF_1327     0    0    0     0      0      0      0      0     0   0
##           PRKD2 EGR1 MAX
## MMRF_1032     0    0   0
## MMRF_1045     0    0   0
## MMRF_1169     0    0   0
## MMRF_1185     0    0   1
## MMRF_1270     0    0   0
## MMRF_1327     0    0   0
```

Test for gene and cytogenetic pairwise interactions

```
genomicData_corr<- all
interactions <- interactionsGenes <- sapply(1:ncol(genomicData_corr), functio
n(i) sapply(1:ncol(genomicData_corr), function(j) {f<- try(fisher.test(genomi
cData_corr[,i], genomicData_corr[,j]), silent=TRUE); if(class(f)=="try-error"
) 0 else ifelse(f$estimate>1, -log10(f$p.val),log10(f$p.val))} ))
oddsRatio <- oddsGenes <- sapply(1:ncol(genomicData_corr), function(i) sapply
(1:ncol(genomicData_corr), function(j) {f<- try(fisher.test(genomicData_corr[
,i] + .5, genomicData_corr[,j] +.5), silent=TRUE); if(class(f)=="try-error")
f=NA else f$estimate} ))
diag(oddsRatio) <- NA
colnames(oddsRatio) <- rownames(oddsRatio) <- colnames(interactions) <- rowna
mes(interactions) <- colnames(genomicData_corr)
oddsRatio[10^-abs(interactions) > 0.05] = 1
oddsRatio[oddsRatio<1e-3] = 1e-4
```

```r
oddsRatio[oddsRatio>1e3] = 1e4
logOdds=log10(oddsRatio)

par(bty="n", mgp = c(2,.5,0), mar=rep(6,4)+.1, las=2, tcl=-.33)
par(mar=c(10,10,5,5))
m <- nrow(oddsRatio)
n <- ncol(oddsRatio)
o = c(1,11,7,3,4,9,6,10,2,5,8,12:m)#h$order#c(h$order,(length(h$order) +1):nc
ol(interactions))
r <- reorder(log10(oddsRatio),o)
r[lower.tri(r)] <- NA
image(x=1:n, y=1:m, r, col=brewer.pal(9,"PiYG"), breaks = c(-4:0-.Machine$dou
ble.eps,0:4), xaxt="n", yaxt="n", xlab="",ylab="", xlim=c(0, n+4), ylim=c(0,
n+4))
r <- reorder(log10(oddsRatio),o)
r[upper.tri(r)] <- NA
mtext(side=2, at=1:n, colnames(oddsRatio)[o], font=ifelse(grepl('[[:lower:]]'
,colnames(oddsRatio)[o]),1,3),
      cex=1, las=1)
mtext(side=1, at=1:n, colnames(oddsRatio)[o], font=ifelse(grepl('[[:lower:]]'
,colnames(oddsRatio)[o]),1,3),
      cex=1, las=2)
abline(h=0:n+.5, col="white", lwd=.5)
abline(v=0:n+.5, col="white", lwd=.5)
text(x=n/2, y=m+1, "Genetic interactions", pos=3, cex=2)
q <- p.adjust(10^-abs(reorder(interactions,o)), method="BH")
p <- p.adjust(10^-abs(reorder(interactions,o)), method="holm")
w = arrayInd(which(q < .1), rep(m,2))
points(w, pch=".", col="white", cex=1.5)
w = arrayInd(which(p < .05), rep(m,2))
points(w, pch="*", col="white")
image(y = 1:8 +6, x=rep(n,2)+c(2,2.5)+1, z=matrix(c(1:8), nrow=1), col=brewer
.pal(8,"PiYG"), add=TRUE)
axis(side = 4, at = seq(1,7) + 6.5,  tcl=-.15, label=10^seq(-3,3), las=1, lwd
=.5)
mtext(side=4, at=10, "Odds ratio", las=3, line=3)
par(xpd=NA)
text(x=n+2.2, y=15, "Correlated", pos=4)
text(x=n+2.2, y=6-.2, "Exclusive", pos=4)
points(x=rep(n,2)+3.5, y=1:2, pch=c("*","."))
image(x=rep(n,2)+c(2,3)+1, y=(3:4) -0.5, z=matrix(1), col=brewer.pal(3,"BrBG"
), add=TRUE)
mtext(side=4, at=1:3, c("P < 0.05", "Q < 0.1", "Not sig."), line=0.2)
```

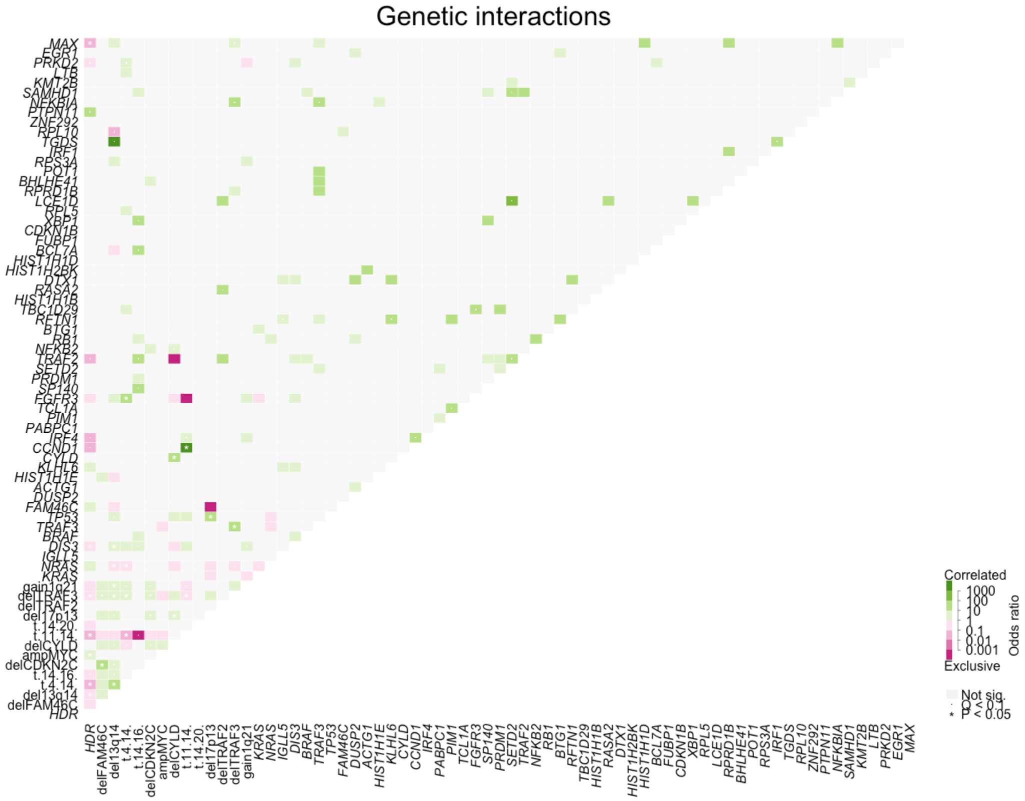

Hierachical Dirichlet Process

```
n<- ncol(genomicData)
shape<- 1
invscale<- 1
hdp<- hdp_init(ppindex=0, #index of the parent DP for initial DP
               cpindex=1, #index of alphaa and alphab for initial DP
               hh=rep(1/n,n), #params for base distn (uniform Dirichlet)
               alphaa=shape,
               alphab=invscale)

hdp<- hdp_adddp(hdp,
                numdp=nrow(genomicData),
                pp=1,
                cp=1)

hdp<- hdp_setdata(hdp= hdp,dpindex=1:nrow(genomicData)+1,data=genomicData)
hdp<- dp_activate(hdp,1:(nrow(genomicData)+1),10)

#chlist <- vector("list", 4)
```

```r
#for (i in 1:4){
#  chlist[[i]] <- hdp_posterior(hdp,
#                                burnin=50000,
#                                n=200,
#                                space=200,
#                                cpiter=3,
#                                seed=i*1e4)
#}
#mut_example_multi <- hdp_multi_chain(chlist)

mut_example_multi<- readRDS("mut_example_multi_chain_MM_50000_69.R") #### upl
oad hdp post 4 chains and burnin 50000)

par(mfrow=c(2,2), mar=c(4, 4, 2, 1))
p1 <- lapply(chains(mut_example_multi), plot_lik, bty="L", start=50000)
```

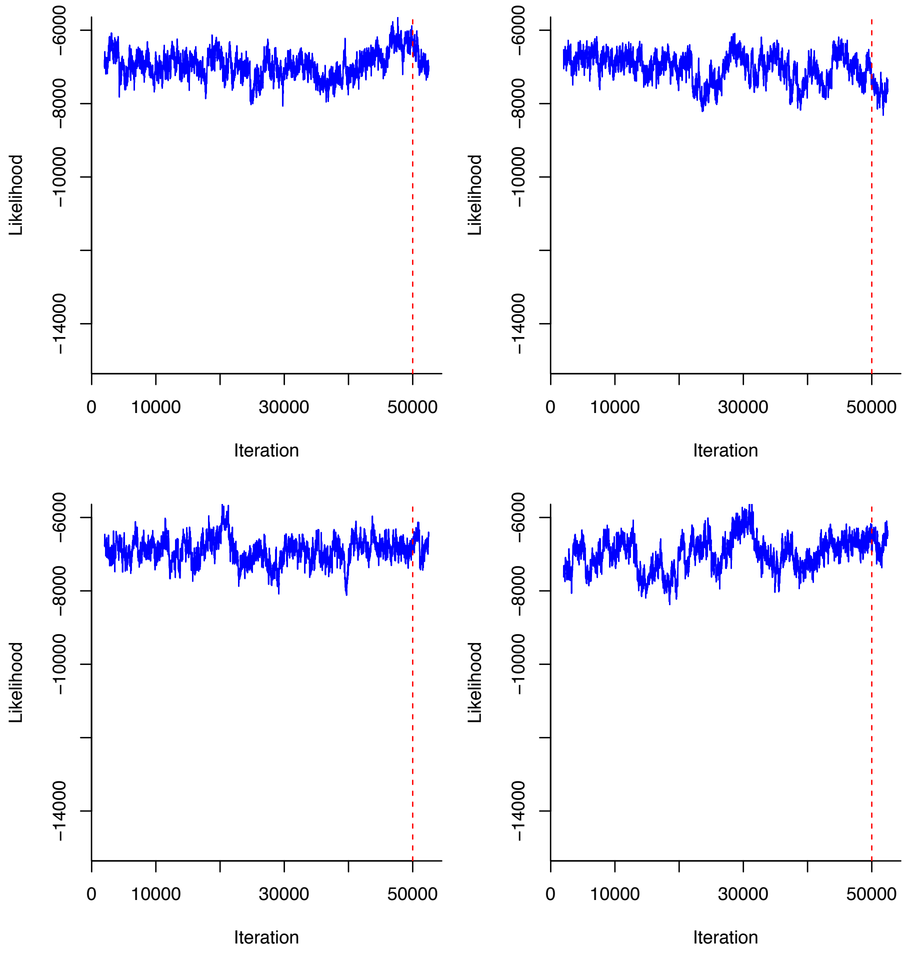

```r
p2 <- lapply(chains(mut_example_multi), plot_numcluster, bty="L")
```

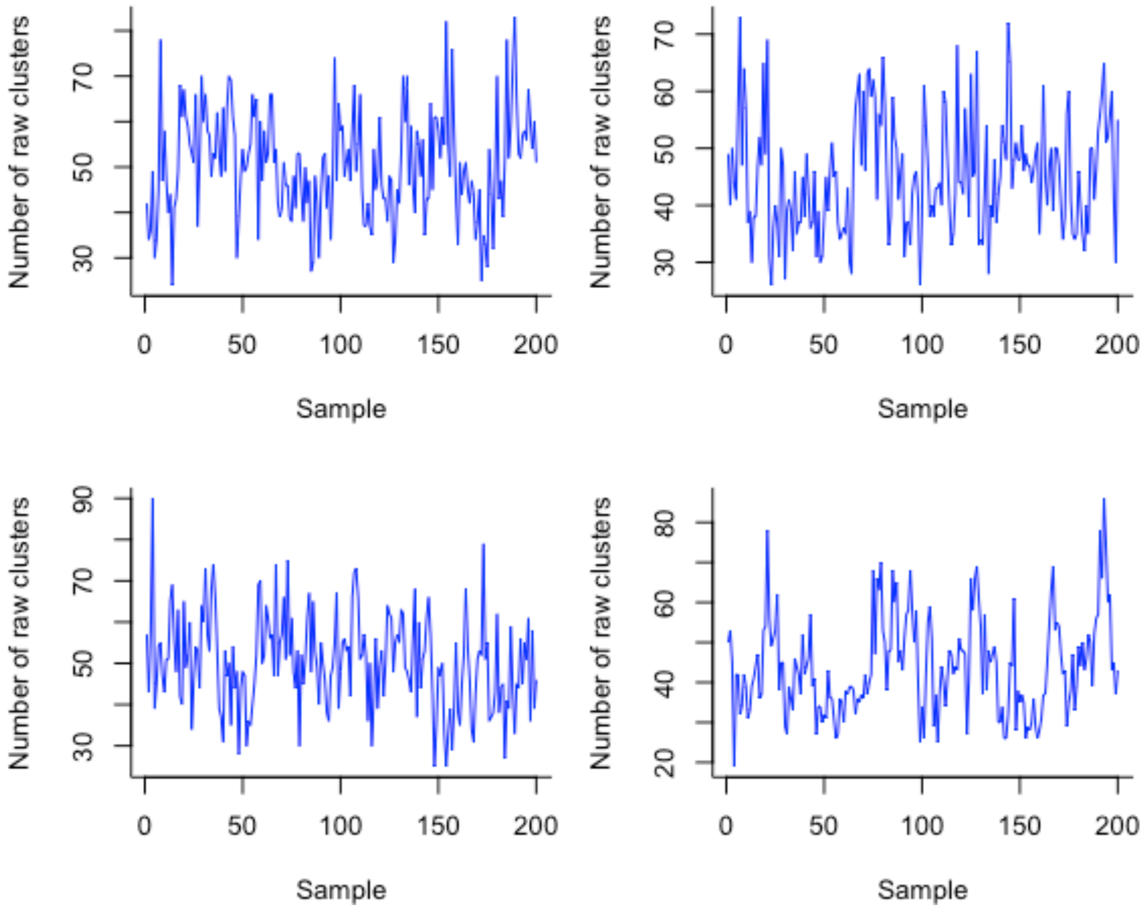

```
p3 <- lapply(chains(mut_example_multi), plot_data_assigned, bty="L")
```

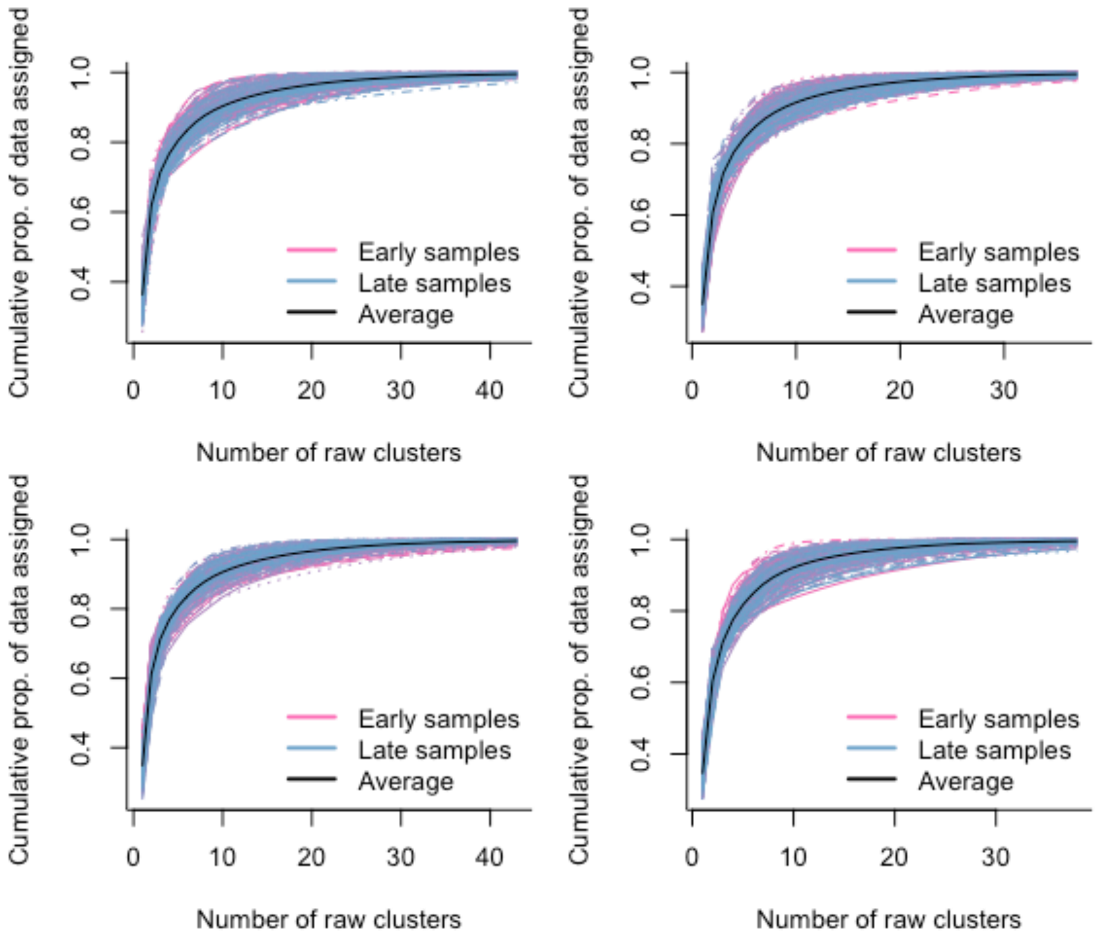

Extract and Described Components

```
#quick_chain_v2 <- hdp_extract_components(mut_example_multi, cos.merge = 0.8, min.sample = 3)

quick_chain_v2<- readRDS("mut_example_multi_chain_MM_50000_6_post_extraction.R")  #### upload extraction component

par(mfrow=c(1,1), mar=c(3, 2, 2, 1))
plot_comp_size(quick_chain_v2, bty="L", lab=c(3, 5, 7))
```

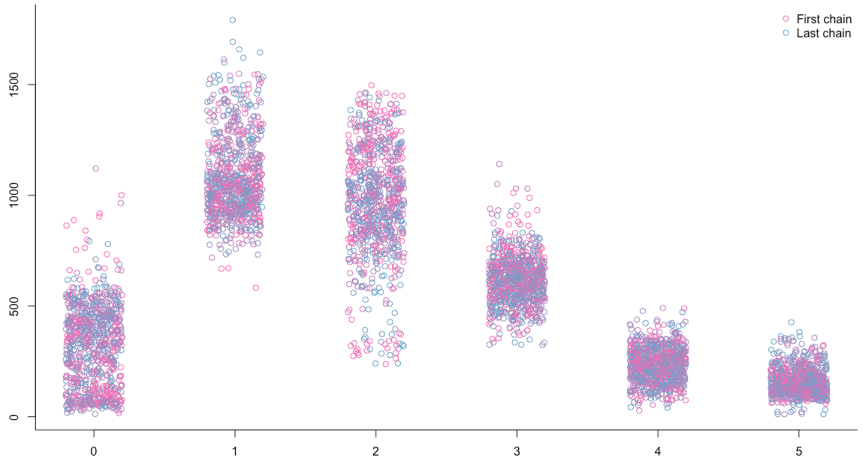


```
par(mfrow=c(3,2), mar=c(7, 2, 2, 1))
plot_comp_distn(quick_chain_v2,  col="skyblue3", cat_names = colnames(genomicData))
```

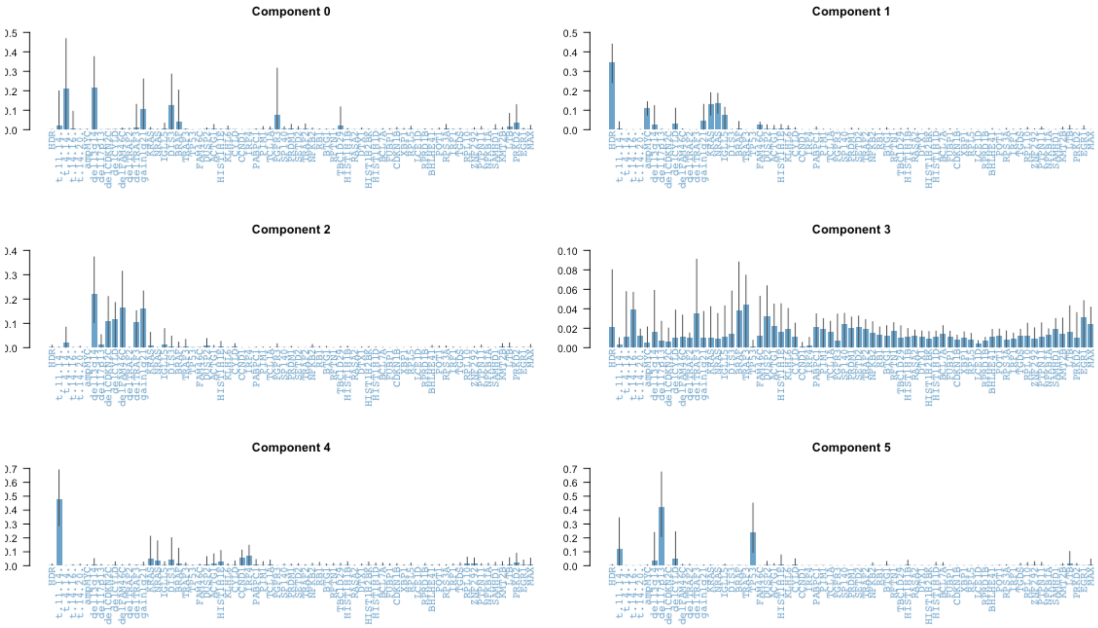

```
par(mfrow=c(1,1), mar=c(3, 2, 2, 1))
plot_dp_comp_exposure(quick_chain_v2, dpindices=2:20, main_text="First 50 sam
ples",
                      col=RColorBrewer::brewer.pal(10, "Set3"))
```

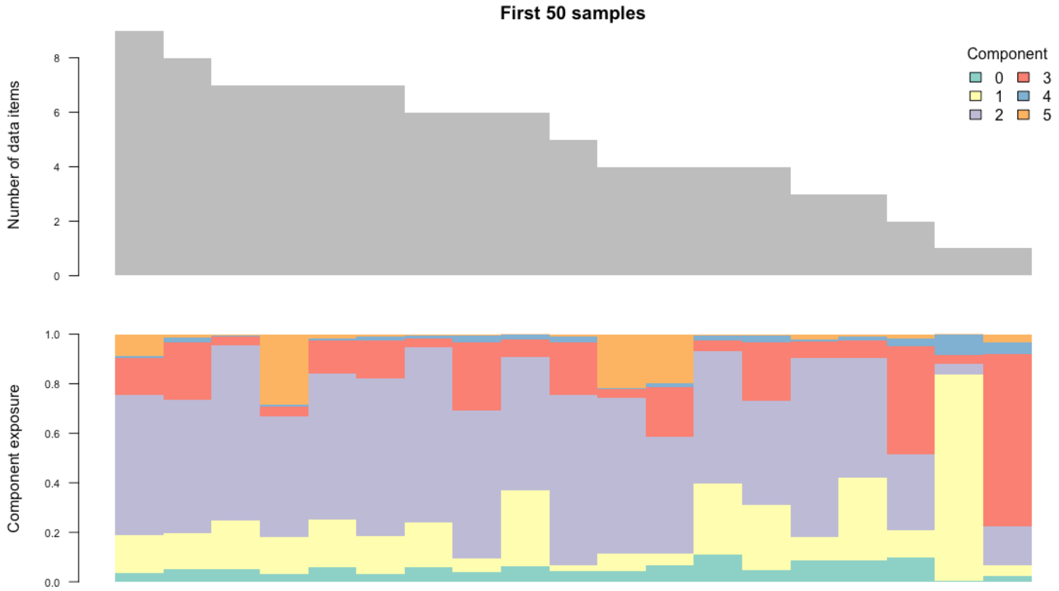

Summary of top genetic contributions to each cluster

```
x<-((quick_chain_v2@comp_dp_distn))
kk<- x[["mean"]]
rownames(genomicData)<- rownames(all)
rownames(kk)<-c("offeset",row.names(genomicData))
posteriorProbability<- t(kk) ##### contribution of each component for each pa
tient
posteriorMeans<- t(comp_categ_distn(quick_chain_v2)[[1]])
rownames(posteriorMeans)<- colnames(genomicData)
genes<- apply(posteriorMeans,2,function(x)paste(ifelse(x>0.10,rownames(poster
iorMeans),"")[order(x,decreasing= TRUE)[1:5]],
                                                collapse=";"))
data.frame(Prob=rowMeans(t(kk), na.rm = T),genes) #### summary of main driver
s for each cluster

##         Prob                                             genes
## 0 0.09111714                  del13q14;t.4.14.;DIS3;gain1q21;
## 1 0.35937728                               HDR;NRAS;KRAS;ampMYC;
## 2 0.26105119 del13q14;delFAM46C;gain1q21;delCYLD;delCDKN2C
## 3 0.16401906                                                ;;;;
## 4 0.08069367                                        t.11.14.;;;;
## 5 0.04374166                            del17p13;TP53;t.11.14.;;
```

Patient class assignment probabilities

```
posteriorProbability<-t(kk[complete.cases(kk),])
dpClass<- factor(apply(posteriorProbability, 2, which.max)-1)
par(mfrow=c(1,1), mar=c(10,5,5,5))
plot(seq(0,1,l=ncol(posteriorProbability)),sort(apply(posteriorProbability,2,
max)),type='l',ylim=c(0,1),xlab="Fraction$of$patients",ylab="Assignment$proba
bility")
```

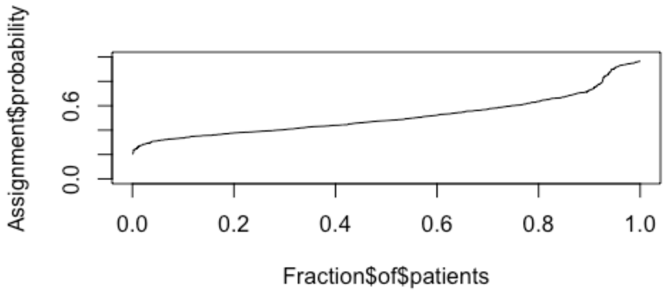


```
par(mfrow=c(1,1), mar=c(3,3,3,3))
boxplot(apply(posteriorProbability,2,max)~ dpClass, col=c(brewer.pal(8,"Dark2
")))
```

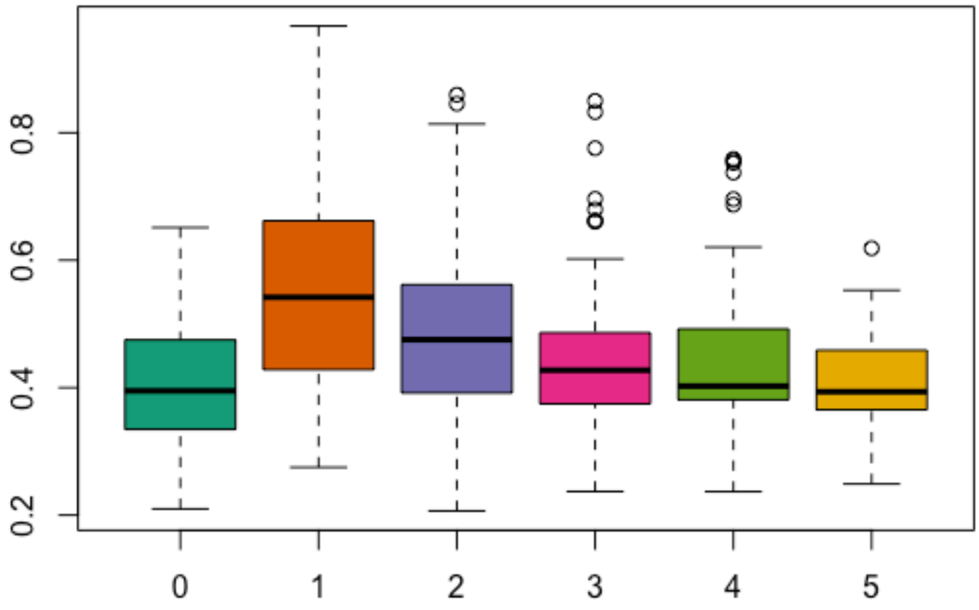

```r
par(mar=c(6,3,1,1)+.1, cex=.8)
o<-  order(colSums(genomicData), decreasing=TRUE)
driverPrevalence<- t(sapply(split(as.data.frame(as.matrix(genomicData)),dpCla
ss),colSums)[o,])
```

```
## Warning in split.default(x = seq_len(nrow(x)), f = f, drop = drop, ...):
## data length is not a multiple of split variable
```

```r
par(mar=c(10,3,1,1)+.1, cex=.8)
b<- barplot(driverPrevalence,col=c(brewer.pal(8,"Dark2")),las=2,legend=TRUE,b
order=NA,args.legend=list(border=NA),  names.arg=rep("",ncol(genomicData)), y
lab = "Probability")
abline(h=seq(100,500,100), col="white")
rotatedLabel(b, labels=colnames(genomicData)[o])
```

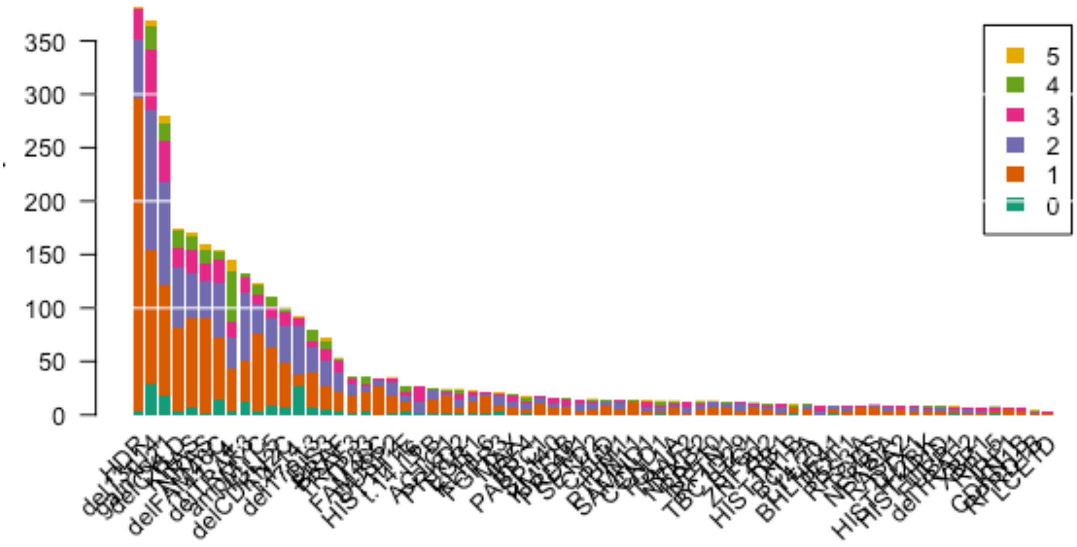

Generate Heatmap of MM drivers: SNVs, CNAs and SVs

```r
cluster<- as.data.frame(dpClass)
cluster$sample<- rownames(cluster)
genomicData2<- as.data.frame(genomicData)
genomicData2$sample<- rownames(genomicData2)
genomicData2$sample<- rownames(genomicData2)
final<- merge(cluster[-1,  ], genomicData2, by="sample")
final[2:ncol(final)]<-apply(final[2:ncol(final)], 2,function(x){as.numeric(as
.character(x))})
rownames(final)<- final$sample
final<- as.data.frame.matrix(final[complete.cases(final),])
```

```r
final<-final[order(final$dpClass, final$HDR, final$t.11.14., final$t.4.14., f
inal$del13q14, final$del17p13),]
annotation_col<- as.data.frame(paste("cluster",final[,2], sep=""))
annotation_col[,1]<-as.factor(annotation_col$`paste("cluster", final[, 2], se
p = "")`)
colnames(annotation_col)[1]<-"cluster"
rownames(annotation_col)<- rownames(final)
mycol_plus<- c(brewer.pal(8,"Dark2"),brewer.pal(6,"Set2"))
ann_colors = list(
  cluster=c("cluster0"=mycol_plus[1], "cluster1"=mycol_plus[2], "cluster2"= m
ycol_plus[3], "cluster3"=mycol_plus[4],
            "cluster4"=mycol_plus[5],  "cluster5"=mycol_plus[6]))
m2<- final[,-c(1,2)]
colnames(m2)[1]<-"Hyperdiploid"
space_heat<- as.numeric(table(final$dpClass))
space_heat2<- c(space_heat[1],
                space_heat[1] + space_heat[2],
                space_heat[1] + space_heat[2] +space_heat[3],
                space_heat[1] + space_heat[2] +space_heat[3]+space_heat[4],
                space_heat[1] + space_heat[2] +space_heat[3]+space_heat[4] +
space_heat[5])

pheatmap(as.matrix(t(m2)), annotation_col=annotation_col , annotation_colors
=ann_colors, cluster_cols = FALSE, show_colnames = F,
         cluster_rows =  FALSE, border_color = FALSE, legend = F, col=c("grey
80","white","gold3","forestgreen","dodgerblue","darkorchid1","red"),
         gaps_col = space_heat2, annotation_legend=FALSE)
```

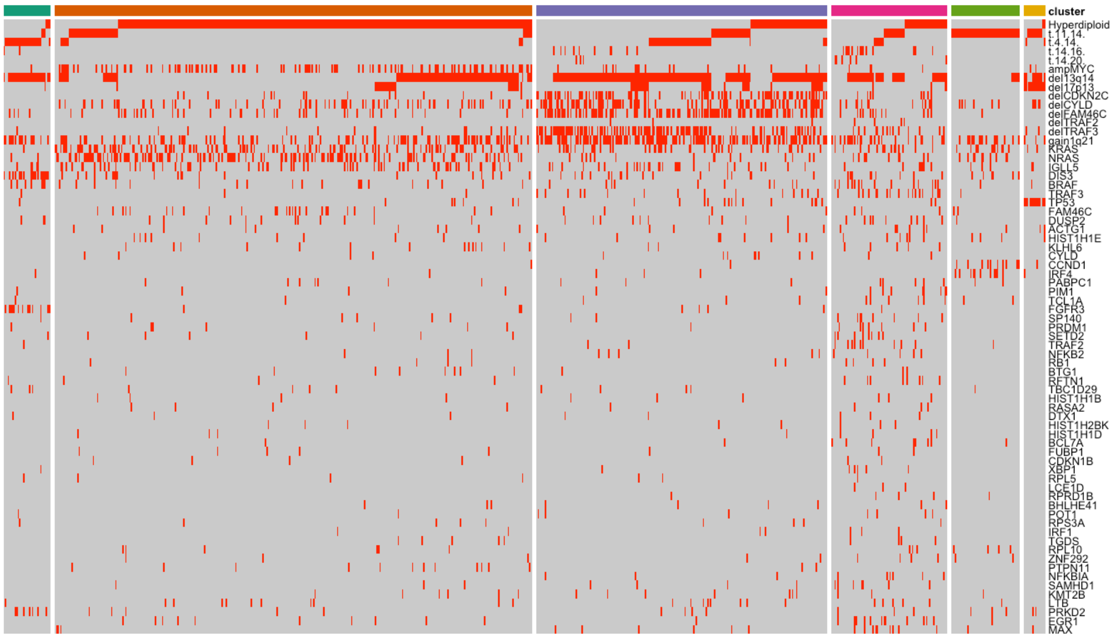

Introduce main Bayesian Network correlation to clean up the hdp clustering

```
m3<- final
m3[,3:ncol(final)][m3[,3:ncol(final)]>1]<-1

#### Mutually exclusive pattern between MAF, HRD, CCND1, MMSET and delTRAF3
maf<- m3[m3$t.14.16.==1 |m3$t.14.20.==1 ,]
maf$dpClass[maf$dpClass==2]<-3

#### BN mutually exclusive pattern between MMSET and HRD, delFAM46C, delCDKN2
C and delCYLD
mmset<- m3[m3$t.4.14.==1,]
mmset$dpClass[mmset$dpClass == 1]<- 0
mmset$dpClass[mmset$dpClass == 3]<- 0
mmset$dpClass[mmset$dpClass == 2]<- 7

### CCND1 mutually exclusive with HRD and low genomic impairment and mutually
exclusive pattern with TRAF3
ccnd1<- m3[m3$t.11.14.==1,]
k2<-ccnd1[ccnd1$dpClass==2,]
ccnd1$dpClass[ccnd1$dpClass == 2]<- 7

#### hyperidploid --> NO CHANGE
HDR<- m3[m3$HDR==1,]

##### Create final data set
sam<- unique(c(mmset$sample, ccnd1$sample, maf$sample))
final_filt<- final[! final$sample %in% sam,]
final_code<- unique(rbind.data.frame(final_filt,mmset, ccnd1, maf))
final2<- merge(genomicData2, final_code[,c(1:2)], by="sample")
rownames(final2)<- final2$sample

### Rename clusters
final2$dpClass[final2$dpClass == 0]<- "Cluster 6"
final2$dpClass[final2$dpClass == 1]<- "Cluster 2"
final2$dpClass[final2$dpClass == 2]<- "Cluster 1"
final2$dpClass[final2$dpClass == 3]<- "Cluster 7"
final2$dpClass[final2$dpClass == 4]<- "Cluster 3"
final2$dpClass[final2$dpClass == 5]<- "Cluster 4"
final2$dpClass[final2$dpClass == 7]<- "Cluster 5"

annotation_col<- as.data.frame(final2[,ncol(final2)])
colnames(annotation_col)[1]<-"cluster"
rownames(annotation_col)<- rownames(final2)
mycol_plus<- c(brewer.pal(8,"Dark2"),brewer.pal(6,"Set2"))
ann_colors = list(cluster=c("Cluster 1"=mycol_plus[1], "Cluster 2"=mycol_plus
[2], "Cluster 3"= mycol_plus[3], "Cluster 4"=mycol_plus[4],
                            "Cluster 5"=mycol_plus[5],  "Cluster 6"=mycol_plu
```

```
s[6],  "Cluster 7"=mycol_plus[7]))
#### new heatmap
final2<- final2[order(final2$dpClass),]
m2<- final2[,-c(1,ncol(final2))]
colnames(m2)[1]<-"Hyperdiploid"
space_heat<- as.numeric(table(final2$dpClass))
space_heat2<- c(space_heat[1],
                space_heat[1] + space_heat[2],
                space_heat[1] + space_heat[2] +space_heat[3],
                space_heat[1] + space_heat[2] +space_heat[3]+space_heat[4],
                space_heat[1] + space_heat[2] +space_heat[3]+space_heat[4] +
space_heat[5],
                space_heat[1] + space_heat[2] +space_heat[3]+space_heat[4] +
space_heat[5]+ space_heat[6])


pheatmap(as.matrix(t(m2)), annotation_col=annotation_col , annotation_colors
=ann_colors, cluster_cols = FALSE, show_colnames = F,
         cluster_rows =  FALSE, border_color = FALSE, legend = F, col=c("grey
80","white","gold3","forestgreen","dodgerblue","darkorchid1","red"),
         gaps_col = space_heat2, annotation_legend=FALSE)
```

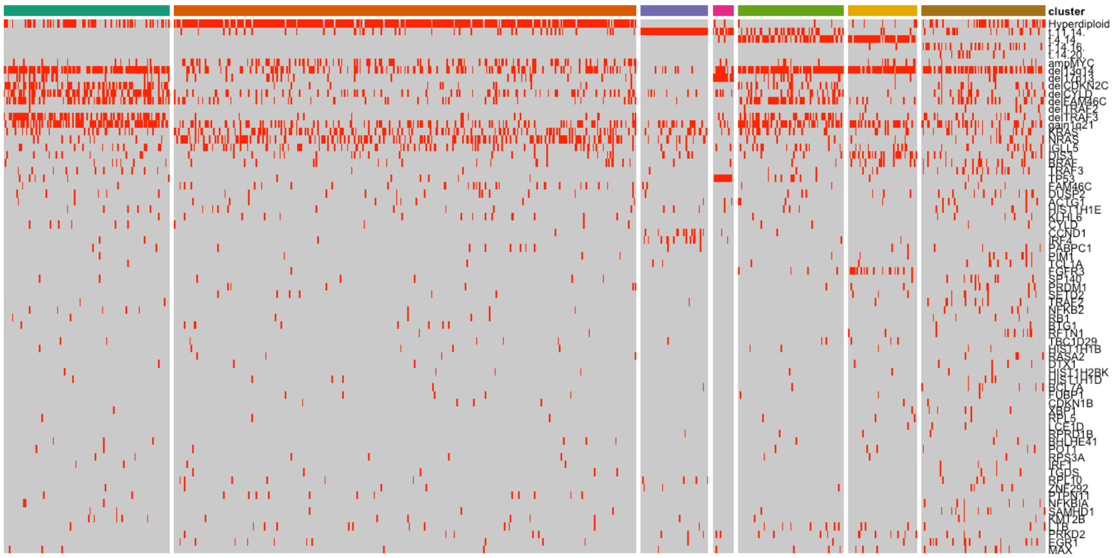

Survival Analysis

```
clin<- read.delim("clinical_commpass.txt", sep="\t", stringsAsFactors = F) ##
# upload clinical information
colnames(clin)[1]<- "sample"
test_clin<- merge(final2, clin, by="sample")

### progression free survival

par(mfrow=c(1,2))
```

```r
plot(survfit(Surv(pfs1cdy,censpfs1) ~ dpClass, data=test_clin), lty = 1, lwd
=2 , mark.time = TRUE,  ylab = "Probability",
     xlab = "Time (Days)", cex.axis = 1.5, cex.lab = 1.5, col=c("dodgerblue3"
,"chartreuse3","brown3","purple2","black","grey80", "forestgreen"))
legend("bottomright", legend=sort(unique(test_clin$dpClass)), col=c("dodgerbl
ue3","chartreuse3","brown3","purple2","black","grey80", "forestgreen"),bty =
"n", lty=1, lwd=2, cex=1, pt.cex=0.5,
       inset=c(+0.1,0.0), x.intersp = 0.5)

### overall survival

plot(survfit(Surv(oscdy,censos) ~ dpClass, data=test_clin), lty = 1, lwd =2 ,
mark.time = TRUE,  ylab = "Probability",
     xlab = "Time (Days)", cex.axis = 1.5, cex.lab = 1.5, col=c("dodgerblue3"
,"chartreuse3","brown3","purple2","black","grey80", "forestgreen"))
legend("bottomright", legend=sort(unique(test_clin$dpClass)), col=c("dodgerbl
ue3","chartreuse3","brown3","purple2","black","grey80", "forestgreen"),bty =
"n", lty=1, lwd=2, cex=1, pt.cex=0.5,
       inset=c(+0.1,0.0), x.intersp = 0.5)
```

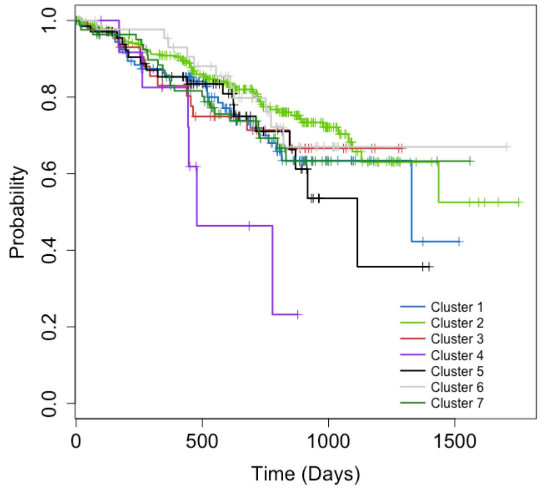

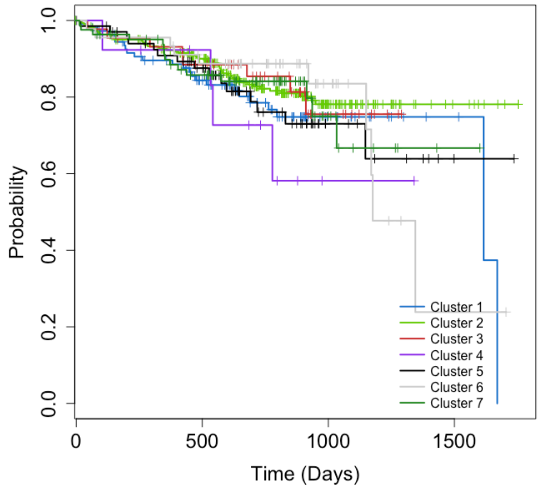

Supplement: Supplementary file 8 — Supplementary Software 1 [file 41467_2019_11680_MOESM8_ESM.pdf]
