# Supplementary material for: Genomic landscape and chronological reconstruction of driver events in multiple myeloma
Source: Nat Commun. 2019 Aug 23;10:3835. doi: 10.1038/s41467-019-11680-1 (PMC6707220; doi:10.1038/s41467-019-11680-1)
Supplement: Supplementary file 9 — Supplementary Software 2 [file 41467_2019_11680_MOESM9_ESM.pdf]

Supplementary Software 2

Tree-finding algorithm

```
Initialise:
    s ← (1, 2, ···, k)        // s is a list of nodes (subclones).
procedure FindRoots(s)        // Fill node list r.
    Identify all nodes in s which could be the root node of the tree;
    Place these candidate root nodes in a new list r;
end procedure
procedure FillTreeList(s, r)        // Fill tree list T.
    T ← ();        // T is an empty tree list.
    for each node a in r do
        o ← (0, 0, ···, 0);        // o = (o[1], o[2], ···, o[k]) is a tree
                                    // in which no node has yet
                                    // been assigned a parent.
        U ← (o);        // U is a tree list
        for each node i in s (excluding node a) do
            V ← ();        // V is an empty tree list
            if U is NOT empty then
                for each tree t in U do        // t = (t[1], t[2], ···, t[k]) is a tree.
                    Find every possible parent node of node i in tree t;
                    Save all these possible parent nodes in a list p(i, t);
                    for each node b in p(i, t) do
                        t' ← t;
                        t'[i] ← b;        // specifies that node b is the parent
                                          // of node i.
                        Insert tree t' into list V;
                    end for
                end for
                Delete element t from list U;
                Insert elements of list V into list U;
            end if
        end for
        Insert elements of list U into list T;
    end for
end procedure
```
